# Differences in H3K4me3 and chromatin accessibility contribute to altered T‐cell receptor signaling in neonatal naïve CD4 T cells

**DOI:** 10.1111/imcb.12561

**Published:** 2022-06-20

**Authors:** Jennifer R Bermick, Priya Issuree, Aaron denDekker, Katherine A Gallagher, Donna Santillan, Steven Kunkel, Nicholas Lukacs, Matthew Schaller

**Affiliations:** ^1^ Division of Neonatology, Department of Pediatrics University of Iowa Iowa City IA USA; ^2^ Division of Neonatal‐Perinatal Medicine, Department of Pediatrics Michigan Medicine Ann Arbor MI USA; ^3^ Department of Internal Medicine University of Iowa Iowa City IA USA; ^4^ Department of Vascular Surgery Michigan Medicine Ann Arbor MI USA; ^5^ Department of Obstetrics and Gynecology University of Iowa Iowa City IA USA; ^6^ Department of Pathology Michigan Medicine Ann Arbor MI USA; ^7^ Mary H. Weiser Food Allergy Center Michigan Medicine Ann Arbor MI USA; ^8^ Pulmonary, Critical Care & Sleep Medicine University of Florida Gainesville FL USA

**Keywords:** ATAC‐seq, ChIP‐seq, epigenetics, neonate, T cell, T‐cell receptor

## Abstract

Neonatal CD4^+^ T cells have reduced or delayed T‐cell receptor (TCR) signaling responses compared with adult cells, but the mechanisms underlying this are poorly understood. This study tested the hypothesis that human neonatal naïve CD4^+^ TCR signaling and activation deficits are related to differences in H3K4me3 patterning and chromatin accessibility. Following initiation of TCR signaling using anti‐CD3/anti‐CD28 beads, adult naïve CD4^+^ T cells demonstrated increased CD69, phospho‐CD3ε and interleukin (IL)‐2, tumor necrosis factor‐α (TNF‐α), interferon‐γ and IL‐17A compared with neonatal cells. By contrast, following TCR‐independent activation using phorbol myristate acetate (PMA)/ionomycin, neonatal cells demonstrated increased expression of CD69, IL‐2 and TNF‐α and equivalent phospho‐ERK compared with adult cells. H3K4me3 chromatin immunoprecipitation‐sequencing (ChIP‐seq) and assay for transposase‐accessible chromatin with high‐throughput sequencing (ATAC‐seq) were performed on separate cohorts of naïve CD4^+^ T cells from term neonates and adults, and RNA‐seq data from neonatal and adult naïve CD4^+^ T cells were obtained from the Blueprint Consortium. Adult cells demonstrated overall increased chromatin accessibility and a higher proportion of H3K4me3 sites associated with open chromatin and active gene transcription compared with neonatal cells. Adult cells demonstrated increased mRNA expression of the TCR‐associated genes *FYN*, *ITK*, *CD4*, *LCK* and *LAT*, which was associated with increased H3K4me3 at the *FYN* and *ITK* gene loci and increased chromatin accessibility at the *CD4*, *LCK* and *LAT* loci. These findings indicate that neonatal TCR‐dependent defects in activation are epigenetically regulated and provide a potentially targetable mechanism to enhance neonatal CD4^+^ T‐cell responses.

## Graphical abstract

This study found that human neonatal naïve CD4^+^ T cells have a T‐cell receptor–dependent defect in activation. This activation defect is associated with decreased mRNA expression of several T‐cell receptor signaling–associated genes in neonatal cells, with decreased H3K4me3 enrichment and/or decreased chromatin accessibility at these gene loci. This suggests that the neonatal defect in CD4^+^ T‐cell receptor signaling is epigenetically regulated.

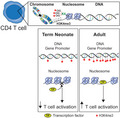

## INTRODUCTION

Infections are the leading cause of morbidity and mortality during the neonatal period, and nearly 1 million neonates die of an infection each year worldwide.^1^ Neonatal infection risk is often attributed to “immaturity” of the immune system. Classifying neonatal immune responses as globally “immature” is not entirely accurate as neonatal immune cells can mount adultlike responses under certain contexts.^2^ It has been well described, however, that under most circumstances neonatal innate and adaptive immune cells demonstrate decreased microbial killing, proliferation and proinflammatory cytokine expression that may contribute to their heightened risk of infection.^3^, ^4^ Neonatal CD4^+^ T cells demonstrate altered responses, which have major implications for vaccine development. T helper type 1 (Th1) adaptive immune responses are crucial to mounting effective responses to immunizations and providing long‐term protection against vaccine‐preventable diseases. Unlike adult CD4^+^ T cells, neonatal CD4^+^ T cells are biased toward Th2 [interleukin (IL)‐4, IL‐5, IL‐13 cytokine expression] rather than Th1 [IL‐2, interferon‐γ (IFN‐γ) cytokine expression] type responses.^5^ In addition, naïve neonatal CD4^+^ T cells demonstrate altered T‐cell receptor (TCR) signaling, including decreased IL‐2 and IFN‐γ expression and decreased expression of the cell surface activation marker CD69.^5^, ^6^ Because of this, there are currently few vaccines that can be administered in the first 2 months of life that provide functional antibody responses leading to long‐term disease protection. A better understanding of the development and function of naïve neonatal CD4^+^ T cells and the factors controlling their lack of Th1 differentiation is needed to optimize neonatal vaccine development and delivery.

Neonatal and adult T cells contain the same genetic material, but exhibit vastly different gene expression and functional capabilities.^7^ Epigenetics provides a potential explanation for these differences. Epigenetics involves changes to DNA structure and gene expression that do not influence the underlying genetic code. Many of these changes include DNA methylation or modifications of histone tails. DNA methylation at gene promoters and regulatory elements silences gene transcription, while histone tail modifications can activate or silence gene transcription by influencing chromatin structure and stability and altering how DNA interacts with transcription factors. Compared with adult CD4^+^ T cells, neonatal CD4^+^ T cells demonstrate hypermethylation at the *IFNG* and *FOXP3* promoters and hypomethylation at the *IL4* promoter, which may partially explain their Th2 bias.^8^ The addition of three methyl groups to lysine (K) 4 of histone (H) 3 (H3K4me3) leads to activation of gene transcription where it occurs.^9^ We have previously shown a developmentally related increase in the transcriptionally activating histone modification H3K4me3 at promoter sites of immunologically important monocyte genes, which allowed for increasingly robust inflammatory responses as development progressed from preterm neonate to adult.^10^ Similar findings have been reported in CD8^+^ T cells, where adult cells demonstrate a global increase in H3K4me3 and improved cytotoxicity compared with neonatal cells.^11^ The role of H3K4me3 and chromatin accessibility in age‐related CD4^+^ T‐cell phenotypes has yet to be studied.

We hypothesized that differences in neonatal naïve CD4^+^ TCR signaling and activation were related to differences in H3K4me3 patterning and chromatin accessibility. To test this hypothesis, we employed massively parallel DNA sequencing [chromatin immunoprecipitation‐sequencing (ChIP‐seq)] for the histone tail modification H3K4me3 and an assay for transposase‐accessible chromatin followed by high‐throughput sequencing (ATAC‐seq) to identify global differences in the H3K4me3 landscape and chromatin accessibility between neonatal and adult naïve CD4^+^ T cells. We integrated these results with RNA‐seq data sets and functional assays to demonstrate that neonatal TCR signaling deficits were associated with epigenetic differences at the level of the chromatin.

## RESULTS

### Neonatal naïve CD4 ^+^ T cells demonstrated a TCR‐dependent defect in activation

**Figure 1:**
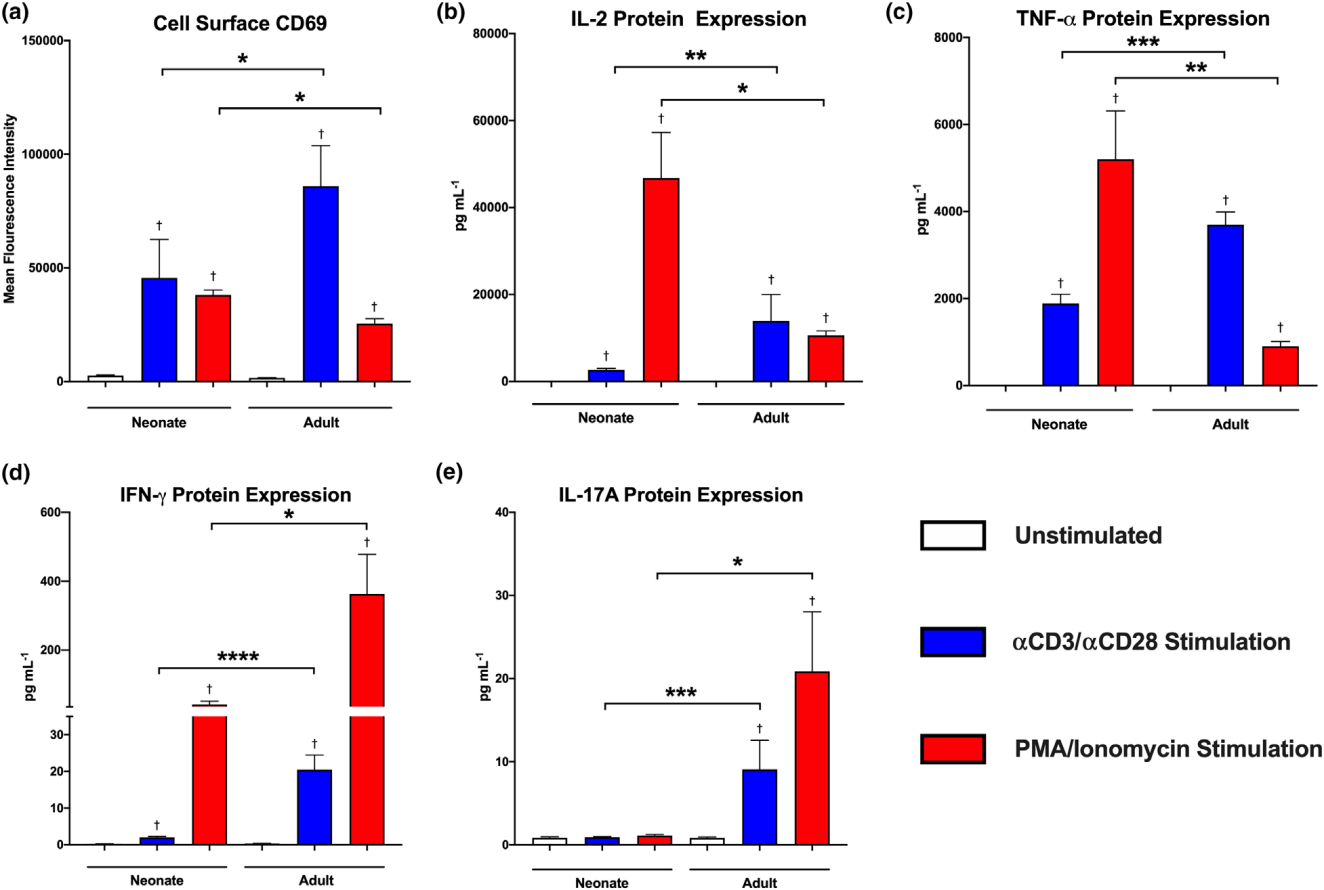
Neonatal naïve CD4^+^ T cells demonstrate decreased markers of activation following T‐cell receptor–dependent stimulation. **(a)** Flow cytometry was used to measure the mean fluorescence intensity of cell surface CD69 on naïve CD4^+^ T cells from adults and healthy term neonates. Mean fluorescence intensity of CD69^+^ receptor expression was quantified in unstimulated cells, 48 h after initiation of T‐cell receptor signaling with anti‐CD3/anti‐CD28 beads or 5 h after T‐cell receptor–independent stimulation with PMA (25 ng mL^−1^) and ionomycin (1 μg mL^−1^). This experiment was performed a total of two times. Neonate unstimulated *n* = 8, Neonate anti‐CD3/anti‐CD28 stimulation *n* = 10, neonate PMA/ionomycin stimulation *n* = 7, adult unstimulated *n* = 17, adult anti‐CD3/anti‐CD28 stimulation *n* = 5, adult PMA/ionomycin stimulation *n* = 5. Boxes represent mean, error bars represent s.e.m. Differences between groups were measured with the Kruskal–Wallis test with Dunn's multiple comparisons test. ^†^ *P* < 0.05 compared with unstimulated control, *P < 0.05. A bead‐based multiplex assay was used to measure cytokine protein levels in cell culture supernatants from naïve CD4^+^ T cells from adults and healthy term neonates. Protein expression was measured 24 h after plating for unstimulated cells, cells stimulated with anti‐CD3/anti‐CD28 beads or cells stimulated with PMA (25 ng mL^−1^) and ionomycin (1 μg mL^−1^). **(b)** IL‐2 protein expression, **(c)** TNF‐α protein expression, **(d)** IFNγ protein expression and **(e)** IL‐17A protein expression. This experiment was performed a total of three times. Neonate unstimulated *n* = 5, neonate anti‐CD3/anti‐CD28 stimulation *n* = 19, neonate PMA/ionomycin stimulation *n* = 5, adult unstimulated *n* = 10, adult anti‐CD3/anti‐CD28 stimulation *n* = 11, adult PMA/ionomycin stimulation *n* = 9. Boxes represent mean, error bars represent s.e.m. Differences between groups were measured with the Kruskal–Wallis test with Dunn's multiple comparisons test. ^†^ *P* < 0.05 compared with unstimulated control, *P < 0.05, **P < 0.01, ***P < 0.001, ****P < 0.0001. IFN, interferon; IL, interleukin; PMA, phorbol myristate acetate; TNF, tumor necrosis factor. [Colour figure can be viewed at wileyonlinelibrary.com]

**Figure 2:**
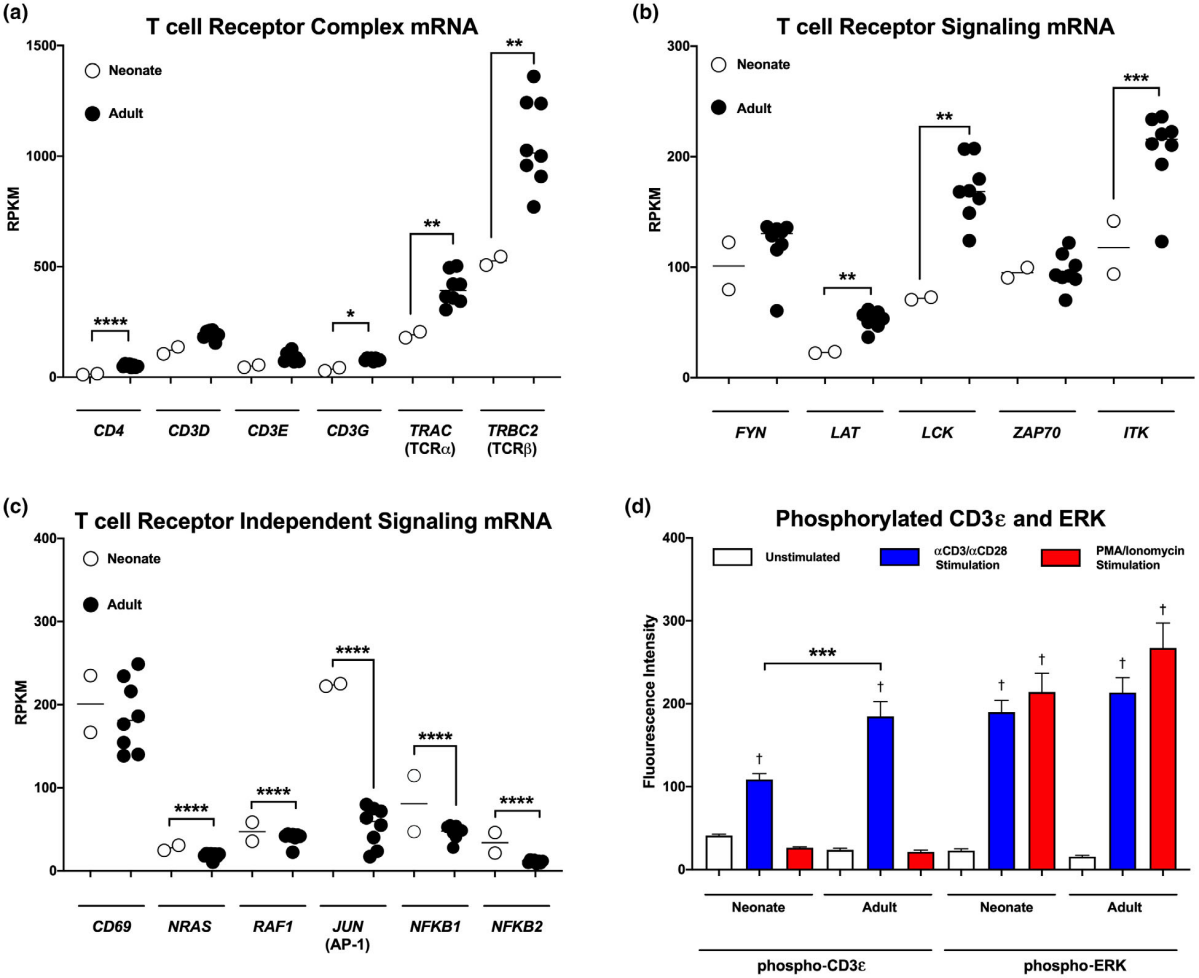
Neonatal naïve CD4^+^ T cells demonstrate decreased T‐cell receptor signaling–related mRNA expression and protein phosphorylation compared with adult cells. Baseline mRNA expression was compared between neonates and adults in **(a)** genes that make up the T‐cell receptor complex, **(b)** downstream T‐cell receptor signaling–specific genes and **(c)** genes involved in T‐cell activation that are not dependent on T‐cell receptor signaling. RNA‐seq data for naïve CD4^+^ T cells from neonates and adults were obtained from the Blueprint Consortium and differential analysis was performed with DESEQ2. mRNA counts were normalized to RPKM. Neonate *n* = 2, adult *n* = 8. *P < 0.05, **P < 0.01, *** *P* < 0.001, ****P < 0.0001. **(d)** Phosphorylation levels of the T‐cell receptor–associated protein CD3ε and the downstream activation–related protein ERK were measured using a bead‐based multiplex assay in neonatal and adult naïve CD4^+^ T cells. Phosphorylation levels were measured immediately following cell purification in unstimulated cells and 30 min following stimulation with either anti‐CD3/anti‐CD28 beads or PMA (25 ng mL^−1^) and ionomycin (1 μg mL^−1^). This experiment was performed a total of three times. Neonate unstimulated *n* = 6, neonate anti‐CD3/anti‐CD28 stimulation *n* = 7, neonate PMA/ionomycin stimulation *n* = 6, adult unstimulated *n* = 6, adult anti‐CD3/anti‐CD28 stimulation *n* = 7, adult PMA/ionomycin stimulation *n* = 6. Boxes represent mean, error bars represent s.e.m. Differences between groups were measured with a one‐way ANOVA with the Holm–Šídák multiple comparisons test. ^†^ *P*‐value < 0.05 compared with unstimulated control, ***P < 0.001. mRNA, messenger RNA; PMA, phorbol myristate acetate; RNA‐seq, RNA sequencing; RPKM, reads per kilobase per million. [Colour figure can be viewed at wileyonlinelibrary.com]

After engagement with an antigen‐presenting cell and initiation of TCR signaling, there is a downstream signaling cascade within the T cell that results in T‐cell activation. This results in upregulation of T‐cell surface markers of activation, including CD69, and expression of multiple cytokines, including IL‐2 and IFN‐γ.^12^ Previous groups have shown that naïve neonatal CD4^+^ T cells have altered TCR signaling, resulting in decreased IL‐2, IFN‐γ and CD69 expression.^5^, ^6^ To confirm these findings in our population, we stimulated naïve adult and neonatal CD4^+^ T cells with anti‐CD3 and anti‐CD28 beads or phorbol myristate acetate (PMA) and ionomycin to engage T‐cell activation in a TCR‐dependent or TCR‐independent manner, respectively. We measured the cell surface marker of T‐cell activation CD69 and cytokine expression following stimulation. Adult and neonatal naïve CD4^+^ T cells had significant upregulation of cell surface CD69 48 h after anti‐CD3/anti‐CD28 stimulation and significant upregulation of cell surface CD69 5 h after PMA/ionomycin stimulation compared with unstimulated cells (Figure 1a). Adult naïve CD4^+^ T cells had increased cell surface CD69 compared with neonatal cells following anti‐CD3/anti‐CD28 stimulation, but decreased cell surface CD69 after PMA/ionomycin stimulation (Figure 1a). Similarly, neonatal naïve CD4^+^ T cells had increased IL‐2 and tumor necrosis factor‐α (TNF‐α) expression following PMA/ionomycin stimulation but decreased IL‐2 and TNF‐α expression following anti‐CD3/anti‐CD28 stimulation compared with adult cells (Figure 1b, c). Meanwhile, adult naïve CD4^+^ T cells expressed higher IFN‐γ and IL‐17A following both anti‐CD3/anti‐CD28 and PMA/ionomycin stimulation compared with the neonatal cells (Figure 1d, e), suggesting a deficit in neonatal cells independent of the method of T‐cell activation. Neither adult nor neonatal cells demonstrated significant expression of the Th2 cytokines IL‐4, IL‐5 or IL‐13 following either anti‐CD3/anti‐CD28 or PMA/ionomycin stimulation (Supplementary figure 1). To investigate mechanisms accounting for differences in T‐cell activation outcomes, we evaluated baseline transcriptional differences between adult and neonatal naïve CD4^+^ T cells using RNA‐seq data sets available through the Blueprint Consortium. Adult naïve CD4^+^ T cells demonstrated increased baseline mRNA expression for several genes necessary for effective TCR‐mediated signaling, including *CD4*, *CD3G*, *TRAC* (TCRα), *TRBC2* (TCRβ), *LAT*, *LCK* and *ITK* (Figure 2a, b). By contrast, neonatal naïve CD4^+^ T cells demonstrated equivalent or increased baseline mRNA expression for genes involved downstream of nonspecific activation, including *CD69*, *NRAS*, *RAF1*, *JUN* (AP‐1), *NFKB1* and *NFKB2* (Figure 2c). We next evaluated differences in phosphorylated proteins critical for successful T‐cell activation following TCR‐dependent or TCR‐independent engagement. Adult naïve CD4^+^ T cells demonstrated increased phosphorylation of CD3ε following anti‐CD3/anti‐CD28 bead crosslinking compared with neonatal cells (Figure 2d). As expected, there was no change in the phosphorylation of CD3ε following stimulation with PMA/ionomycin as this is known to be a TCR‐specific event (Figure 2d). Interestingly, there were no differences in phosphorylated levels of the downstream common activation protein extracellular signal‐regulated kinase (ERK) following either anti‐CD3/anti‐CD28 or PMA/ionomycin stimulation (Figure 2d), although kinetic differences in ERK phosphorylation cannot be ruled out. These data demonstrate that neonatal naïve CD4^+^ T cells respond differentially when engaged in a TCR‐dependent manner compared with adult naïve CD4^+^ T cells but can respond robustly when the TCR is bypassed.

### Adult and neonatal naïve CD4 ^+^ T cells differ in H3K4me3 enrichment

**Figure 3:**
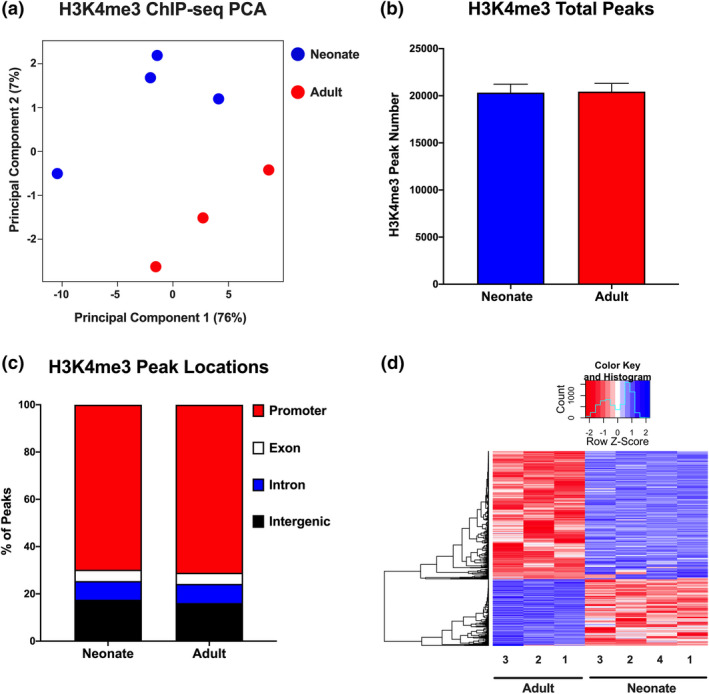
Adult and neonatal naïve CD4^+^ T cells demonstrate differences in H3K4me3 enrichment. **(a)** PCA of naïve CD4^+^ T‐cell ChIP‐seq H3K4me3‐normalized read counts. Visual produced using the program DiffBind. **(b)** Total consensus naïve CD4^+^ T‐cell ChIP‐seq H3K4me3 peaks, defined as peaks that exist in more than one replicate. Boxes represent mean, error bars represent s.e.m. Difference between groups was assessed by the unpaired *t‐*test. **(c)** H3K4me3 ChIP‐seq peaks by location. Promoter locations were defined as 1‐kb upstream and downstream of transcriptional start sites. Difference between groups was assessed by Fisher's exact test. **(d)** Heatmap demonstrating H3K4me3‐binding affinity (measured by differences in read densities between samples) using ChIP‐seq–normalized read counts between adult naïve CD4^+^ T cells and naïve CD4^+^ T cells from healthy term neonates. Visual produced using the program DiffBind. Numbers indicate replicates. ChIP‐seq experiment performed a total of one time. Neonate *n* = 4, adult *n* = 3. ChIP‐seq, chromatin immunoprecipitation sequencing; PCA, principal component analysis. [Colour figure can be viewed at wileyonlinelibrary.com]

**Figure 4:**
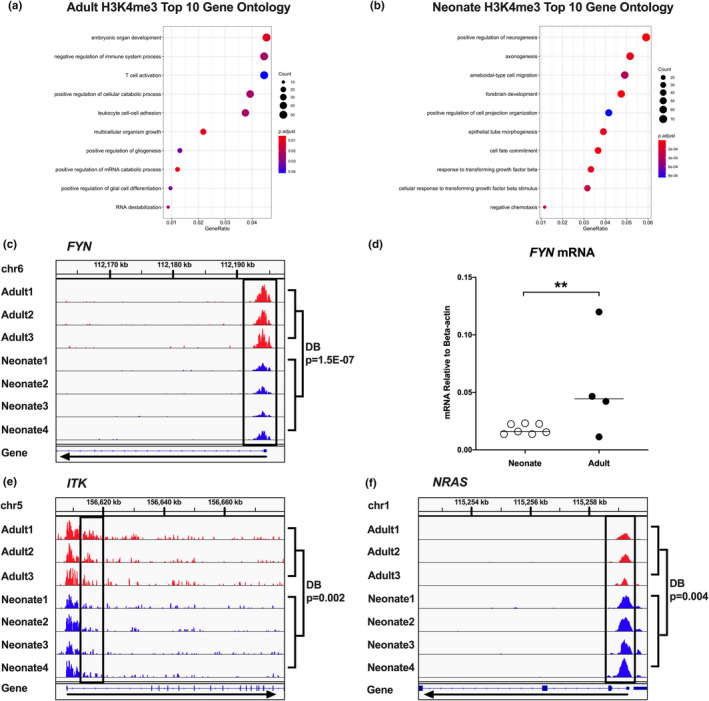
Neonatal and adult naïve CD4^+^ T cells demonstrate differences in H3K4me3 abundance at genes involved in T‐cell receptor signaling and T‐cell activation. **(a)** Top 10 gene ontology pathways with H3K4me3 enrichment in adult naïve CD4^+^ T cells. Visual produced with the program clusterProfiler. **(b)** Top 10 gene ontology pathways with H3K4me3 enrichment in neonatal naïve CD4^+^ T cells. Visual produced with the program clusterProfiler. **(c)** Adult naïve CD4^+^ T cells demonstrate increased H3K4me3 at the *FYN* promoter (FYN proto‐oncogene, Src family tyrosine kinase) based on edgeR differential binding compared with neonatal naïve CD4^+^ T cells. Adult *n* = 3, neonate *n* = 4, number represents replicate. The black rectangle highlights the differential peaks. ChIP‐seq experiment performed a total of one time. The black arrow indicates gene direction. H3K4me3 peaks were visualized using the Integrated Genomics Viewer. **(d)** Unstimulated adult naïve CD4^+^ T cells had increased *FYN* mRNA expression by qPCR. *FYN* mRNA expression was normalized to the housekeeping gene *ACTB* (β‐actin) using the ΔCT method. Adult *n* = 4, neonate *n* = 7. This experiment was performed a total of two times. Individual data points are shown and the bar represents median. Differences between groups were evaluated using the Mann–Whitney test. **P < 0.01. **(e)** Adult naïve CD4^+^ T cells demonstrate increased H3K4me3 at the first intron of the *ITK* gene (IL‐2–inducible T‐cell kinase) compared with neonatal cells based on edgeR differential binding. Adult *n* = 3, neonate *n* = 4, number represents replicate. The black rectangle highlights the differential peaks. ChIP‐seq experiment performed a total of one time. The black arrow indicates gene direction. H3K4me3 peaks were visualized using the Integrated Genomics Viewer. **(f)** Neonatal naïve CD4^+^ T cells demonstrate increased H3K4me3 at the promoter of the *NRAS* gene (NRAS proto‐oncogene, GTPase) compared with adult cells based on edgeR differential binding. Adult *n* = 3, neonate *n* = 4, number represents replicate. The black rectangle highlights the differential peaks. ChIP‐seq experiment performed a total of one time. The black arrow indicates gene direction. H3K4me3 peaks were visualized using the Integrated Genomics Viewer. ChIP, chromatin immunoprecipitation; DB, differentially bound; IL, interleukin; mRNA, messenger RNA; qPCR, quantitative PCR. [Colour figure can be viewed at wileyonlinelibrary.com]

We have previously demonstrated that monocytes gained promoter‐site H3K4me3 as development progressed from preterm neonate to adult and that this gain was associated with differences in cell function.^10^, ^13^ To determine whether T cells experience a similar developmental gain in H3K4me3 that could explain the neonatal naïve CD4^+^ T‐cell defect in TCR signaling, we performed H3K4me3 ChIP‐seq on peripheral blood naïve CD4^+^ T cells from healthy adults and umbilical cord blood naïve CD4^+^ T cells from healthy term neonates. A principal component analysis of normalized read counts demonstrated separate clustering of the adult and neonatal cells (Figure 3a), but equivalent total H3K4me3 peaks and similar global peak locations (Figure 3b, c). Peak locations are detailed in Supplementary tables 1 and 2. A binding affinity analysis (measured by differences in read densities between samples) was then performed to determine sites with statistically significantly differentially bound H3K4me3 between adult and neonatal naïve CD4^+^ T cells. This showed that adult naïve CD4^+^ T cells had distinctly different H3K4me3 peak signal patterns than neonatal naïve CD4^+^ T cells (Figure 3d). A total of 3389 sites had differential H3K4me3 between adult and neonatal naïve CD4^+^ T cells, with 1597 of these sites having increased levels (read density) in adult cells (Supplementary table 3). Both adult and neonatal cells demonstrated H3K4me3 enrichment in gene ontology pathways associated with metabolism and development, including embryonic and nervous system development (Figure 4a, b and Supplementary table 4). However, only adult cells demonstrated H3K4me3 enrichment in pathways associated with T‐cell activation (Figure 4a and Supplementary table 4). Adult naïve CD4^+^ T cells had increased H3K4me3 at the promoter site of the downstream TCR signaling gene *FYN* compared with neonatal cells (Figure 4c). Although there was an increased trend but no statistical difference in *FYN* mRNA expression between adult and neonatal cells in the Blueprint RNA‐seq data set (Figure 2b), we found increased *FYN* mRNA expression in adult naïve CD4^+^ T cells by qPCR that correlated with this difference in H3K4me3 abundance (Figure 4d). We also found that increased expression of the downstream TCR signaling gene *ITK* in adult cells (Figure 2b) was associated with increased H3K4me3 in the first intron of the *ITK* gene locus in adult cells with equivalent H3K4me3 at the promoter (Figure 4e). Similarly, increased mRNA expression of the TCR‐independent activation gene *NRAS* (Figure 2c) was associated with increased promoter‐site H3K4me3 in the neonatal cells (Figure 4f). We next sought to determine whether differences in expression of chromatin‐modifying enzymes were associated with the differences in naïve CD4^+^ T‐cell H3K4me3 between neonates and adults. There are eight H3K4 methyltransferases that perform trimethylation, although not all of these are active in human tissues.^14^ We measured the mRNA expression of seven H3K4 methyltransferases known to be active in human tissues in unstimulated naïve CD4^+^ T cells from adults and healthy term neonates. Adult cells demonstrated increased *KMT2A* (*MLL1*) and *SET1A* mRNA compared with neonatal cells (Supplementary figure 2), but the role these methyltransferases play in H3K4me3 deposition in naïve CD4^+^ T cells warrants further investigation.

### Adult naïve CD4 ^+^ T cells display increased chromatin accessibility and gene transcription at sites of H3K4me3 enrichment

**Figure 5:**
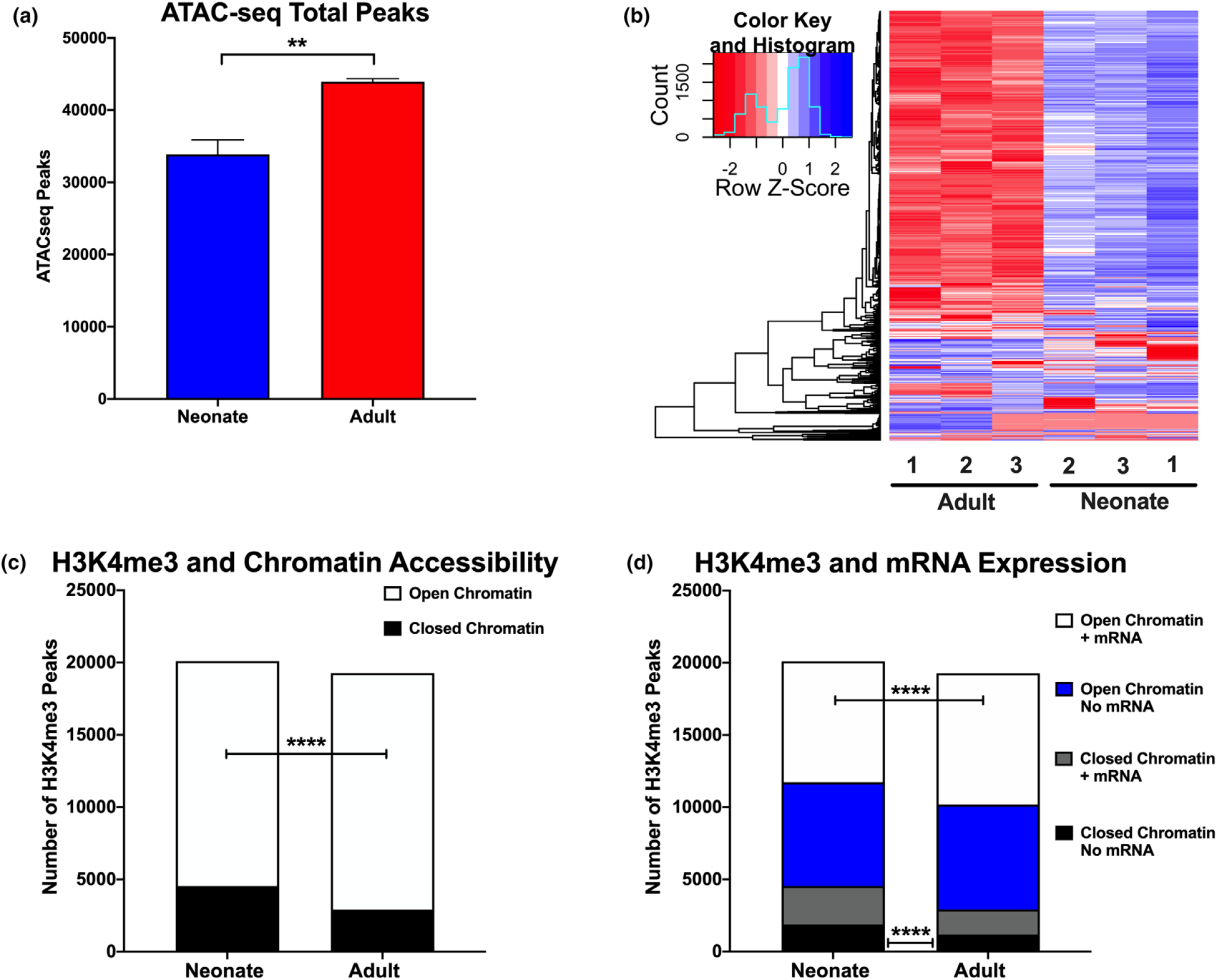
Adult naïve CD4^+^ T cells have more accessible chromatin at sites of H3K4me3 compared with neonatal cells. **(a)** Total consensus naïve CD4^+^ T‐cell ATAC‐seq peaks, defined as peaks that exist in more than one replicate. Adult *n* = 3, neonate *n* = 3. ATAC‐seq experiment performed a total of one time. Boxes represent mean, error bars represent s.e.m. Difference between groups was assessed by the unpaired *t*‐test. **P < 0.01. **(b)** Heatmap demonstrating ATAC‐seq–binding affinity (measured by differences in read densities between samples) using normalized ATAC‐seq read counts between adult naïve CD4^+^ T cells and naïve CD4^+^ T cells from healthy term neonates. Visual produced using the program DiffBind. Numbers indicate replicates. ATAC‐seq experiment performed a total of one time. Neonate *n* = 3, adult *n* = 3. **(c)** Direct comparison of ATAC‐seq and H3K4me3 ChIP‐seq peaks using DiffBind. Open chromatin was defined by the presence of an ATAC‐seq peak. Closed chromatin had no detectable ATAC‐seq peak. ATAC‐seq and ChIP‐seq experiments performed a total of one time. ATAC‐seq neonate *n* = 3, adult *n* = 3. H3K4me3 ChIP‐seq neonate *n* = 4, adult *n* = 3. Difference between groups was assessed by Fisher's exact test. ****P < 0.0001. **(d)** Comparison of mRNA transcription, H3K4me3 enrichment and chromatin accessibility in adult and neonatal naïve CD4^+^ T cells. RNA‐seq data for naïve CD4^+^ T cells from neonates and adults were obtained from the Blueprint Consortium. mRNA counts were normalized to RPKM. mRNA transcription was considered present if RPKM ≥ 1 and absent if < 1. Chromatin was considered open if there was an identifiable ATAC‐seq peak at the location and was considered closed if no peak was present. ATAC‐seq and ChIP‐seq experiments performed a total of one time. ATAC‐seq neonate *n* = 3, adult *n* = 3. H3K4me3 ChIP‐seq neonate *n* = 4, adult *n* = 3. RNA‐seq neonate *n* = 2, adult *n* = 8. Difference between groups was assessed by Fisher's exact test. ****P < 0.0001. ATAC‐seq, assay for transposase‐accessible chromatin with high‐throughput sequencing; ChIP‐seq, chromatin immunoprecipitation‐sequencing, mRNA, messenger RNA; RPKM, reads per kilobase per million. [Colour figure can be viewed at wileyonlinelibrary.com]

While differences in H3K4me3 abundance in adult naïve CD4^+^ T cells were associated with differences in mRNA expression in the TCR signaling genes *FYN* and *ITK*, transcriptional differences in several additional TCR signaling genes could not be explained solely by H3K4me3. As other epigenetic features could dictate the differential TCR responses of adult and neonatal T cells, we also performed an ATAC‐seq using a different set of adult and neonatal naïve CD4^+^ T cells to evaluate the chromatin accessibility patterns surrounding sites of H3K4me3 enrichment.^15^ Adult naïve CD4^+^ T cells had increased global chromatin accessibility and a distinctly different chromatin accessibility pattern (read density) compared with neonatal naïve CD4^+^ T cells (Figure 5a, b). Peak locations are detailed in Supplementary tables 5 and 6 and differentially bound peaks are located in Supplementary table 7. A direct comparison of adult and neonatal ATAC‐seq and H3K4me3 ChIP‐seq peak locations showed that while the total number of H3K4me3 peaks were similar in neonatal and adult CD4^+^ T cells (Figure 3b), H3K4me3 was more likely to be associated with open and accessible chromatin in adult cells (Figure 5c). We next correlated mRNA expression with the presence of H3K4me3 and chromatin accessibility using the Blueprint RNA‐seq data sets. We found that H3K4me3 peaks in adult naïve CD4^+^ T cells were more likely to be associated with open chromatin and active gene transcription while they were more likely to be associated with closed chromatin and no mRNA expression in neonatal cells (Figure 5d). Although H3K4me3 is commonly associated with accessible chromatin and active gene transcription, our data demonstrate that this is less often the case in neonatal naïve CD4^+^ T cells.

### Adult naïve CD4 ^+^ T cells have increased chromatin accessibility at TCR signaling genes

**Figure 6:**
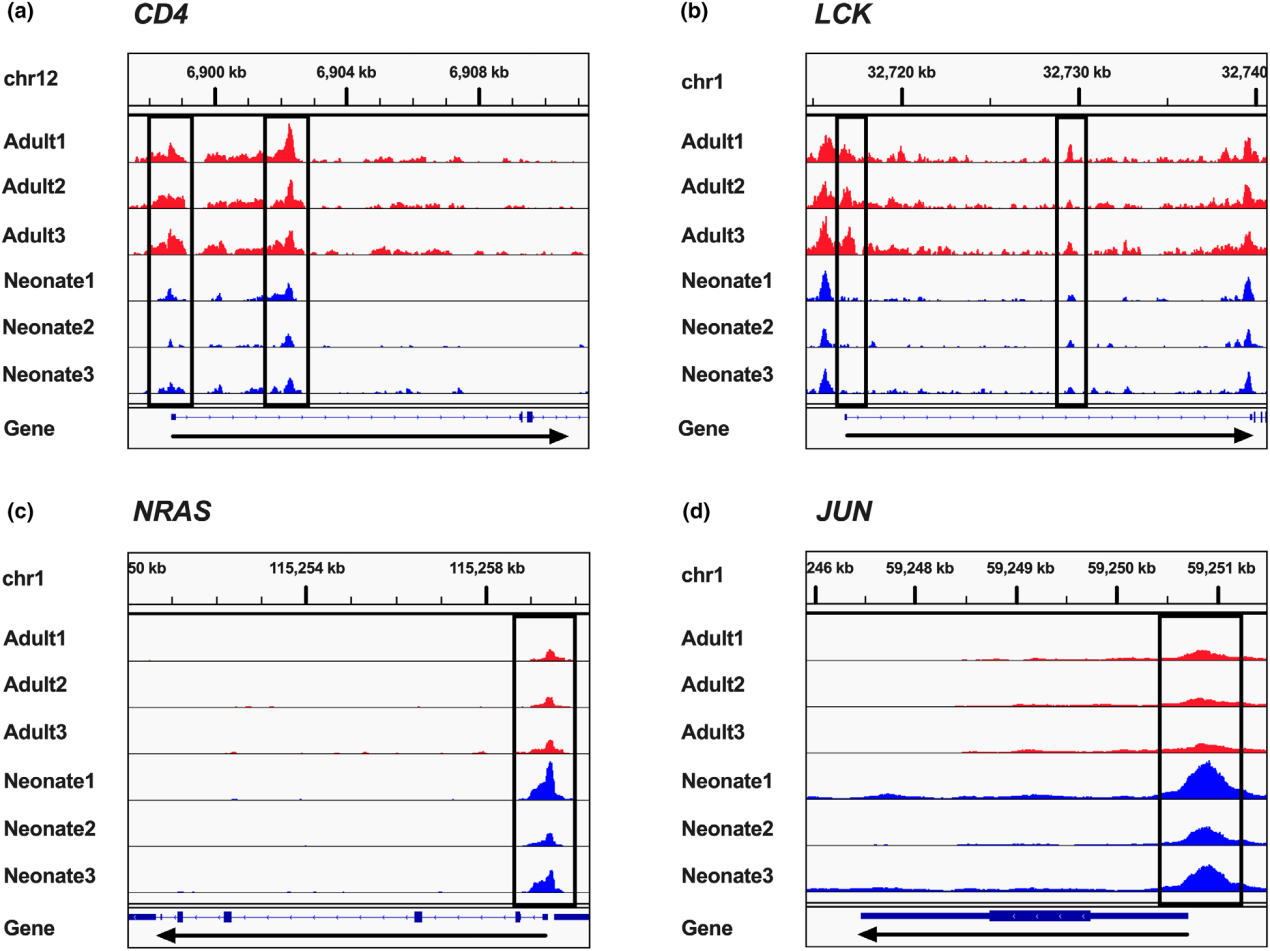
Adult naïve CD4^+^ T cells demonstrate increased chromatin accessibility in T‐cell receptor signaling gene loci, while neonatal naïve CD4^+^ T cells demonstrate enhanced chromatin accessibility in T‐cell receptor–independent activation gene loci. **(a)** Adult naïve CD4^+^ T cells have increased ATAC‐seq peaks at the promoter and first intron of the T‐cell receptor–associated gene *CD4* compared with neonatal cells based on edgeR differential binding. **(b)** Adult naïve CD4^+^ T cells have increased ATAC‐seq peaks at the promoter and first intron of the T‐cell receptor signaling gene *LCK* (LCK proto‐oncogene, Src family tyrosine kinase) compared with neonatal cells based on edgeR differential binding. **(c)** Neonatal naïve CD4^+^ T cells have increased ATAC‐seq peaks at the promoter of the common T‐cell activation gene *NRAS* (NRAS proto‐oncogene, GTPase) compared with adult cells based on edgeR differential binding. **(d)** Neonatal naïve CD4^+^ T cells have increased ATAC‐seq peaks at the promoter of the common downstream T‐cell activation gene *JUN* (JUN proto‐oncogene, AP‐1 transcription factor subunit) compared with adult cells based on edgeR differential binding. Adult *n* = 3, neonate *n* = 3, number represents replicate. The black rectangle highlights the differential peaks. ATAC‐seq experiment performed a total of one time. The black arrow indicates gene direction. ATAC‐seq peaks were visualized using the Integrated Genomics Viewer. ATAC‐seq, assay for transposase‐accessible chromatin with high‐throughput sequencing. [Colour figure can be viewed at wileyonlinelibrary.com]

We next evaluated whether adult and neonatal naïve CD4^+^ T cells had differences in chromatin accessibility at TCR‐specific genes that could explain functional differences following TCR engagement. While there was increased H3K4me3 abundance at the *FYN* and *ITK* gene loci in adult cells (Figure 4c, e), this was not associated with a clear pattern of enhanced chromatin accessibility at these loci in adult cells (Table 1). Adult cells did, however, demonstrate enhanced chromatin accessibility at several TCR signaling–specific gene loci with increased mRNA expression in the adult cells, including *CD4*, *LAT* and *LCK* (Figures 2a, b and 6a, b and Supplementary figure 3a). Similarly, neonatal cells had increased chromatin accessibility at multiple TCR‐independent activation gene loci in the neonatal cells, including *NRAS*, *JUN* (AP‐1), *RAF1*, *NFKB1* and *NFKB2*, that was associated with increased mRNA expression (Figures 2c and 6c, d and Supplementary figure 3b–d). This suggests that differences in chromatin accessibility contribute to the altered neonatal T‐cell activation responses.

**Table 1:** Differentially bound ATAC‐seq peaks in T‐cell activation pathways between adult and neonatal naïve CD4^+^ T cells

Gene	Increased chromatin accessibility	Location	ATAC‐seq differential binding *P*‐value
T‐cell receptor–specific activation			
*CD4*	Adult	Promoter	0.0003
	Adult	Intron 1	0.005
*FYN*	Adult	Promoter	0.006
	Adult	Exon 8	0.01
	Adult	Intron 9	0.01
	Neonate	Promoter	2.6 × 10^−7^
	Neonate	5′ UTR	0.008
	Neonate	Intron 1	3.2 × 10^−6^
*ITK*	Neonate	Intron1	0.003
*LAT*	Adult	Promoter	0.006
	Adult	Intron 2	0.0001
*LCK*	Adult	Promoter	2.3 × 10^−10^
	Adult	Intron 1	0.003
*ZAP70*	Adult	Promoter	0.009
	Adult	Intron 2	0.003
	Adult	3′ UTR	0.002
T‐cell receptor–independent activation			
*CD69*	Neonate	Promoter	7.8 × 10^−12^
	Neonate	Intron 1	0.001
*IKBKB*	Adult	Intron 5	5.6 × 10^−5^
	Adult	3′ UTR	0.008
	Neonate	Promoter	0.02
*IKBKG*	Neonate	Promoter	0.0006
*JUN*	Neonate	Promoter	2.1 × 10^−10^
	Neonate	3′ UTR	2.4 × 10^−6^
*MAP2K1* (MEK1)	Neonate	Promoter	0.0006
*MAP2K2* (MEK2)	Adult	Intron 3	1.1 × 10^−5^
*MAPK1* (ERK2)	Adult	Intron 1	0.0009
*NFKB1*	Neonate	Promoter	1.45 × 10^−5^
	Neonate	Intron 1	4.1 × 10^−5^
	Neonate	Intron 2	0.005
	Neonate	Intron 4	0.002
	Neonate	Intron 5	7.1 × 10^−6^
	Neonate	Intron 14	0.003
	Neonate	Intron 15	0.0001
*NFKB2*	Neonate	Promoter	0.0002
*NRAS*	Neonate	Promoter	3.2 × 10^−5^
*RAF1*	Neonate	Promoter	3.1 × 10^−7^

ATAC‐seq, assay for transposase‐accessible chromatin with high‐throughput sequencing; UTR, untranslated region.

### Adult and neonatal naïve CD4 ^+^ T cells demonstrate differences in chromatin accessibility at key transcription factors associated with Th1, Th2 and Th17 cellular programs

**Figure 7:**
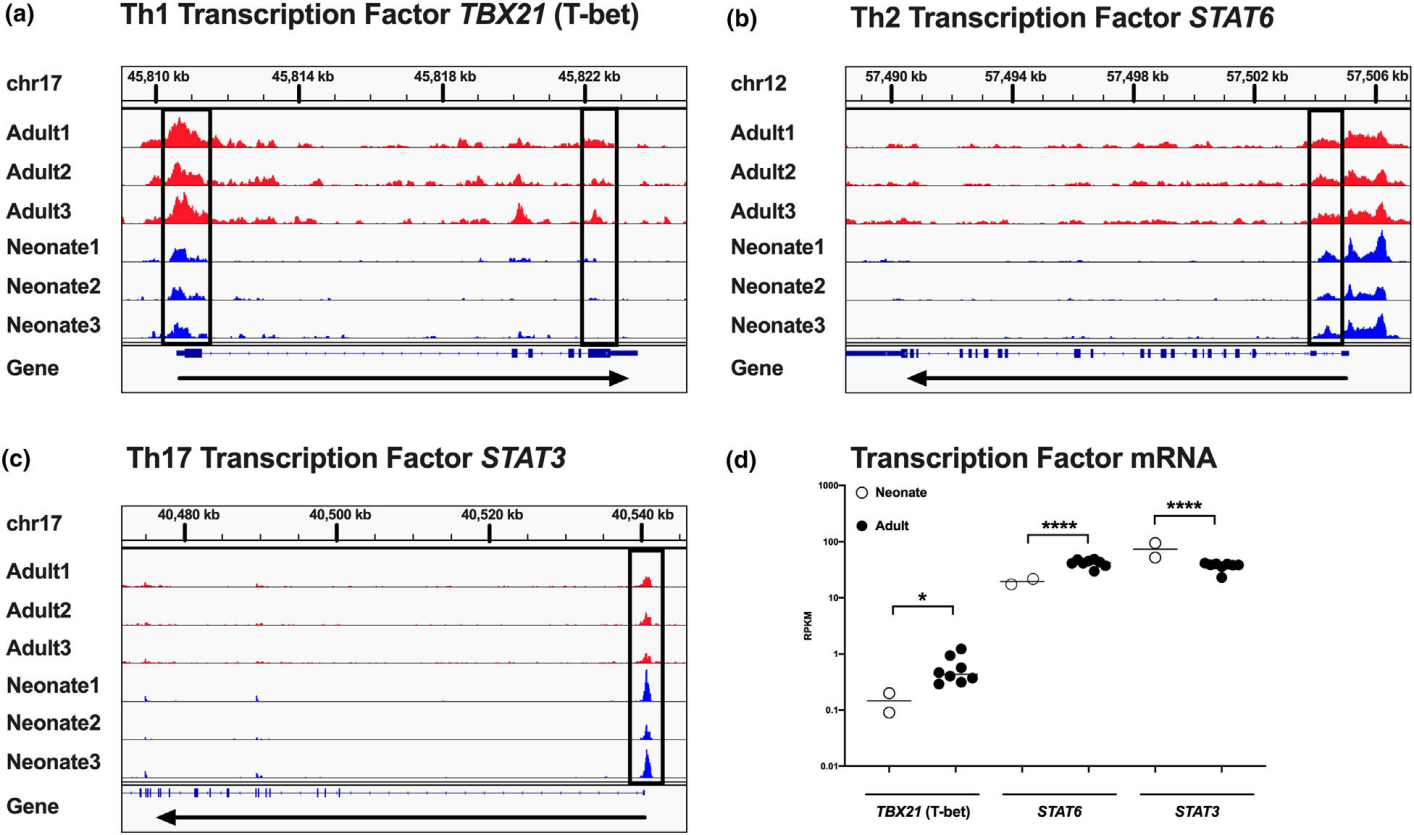
Neonatal and adult naïve CD4^+^ T cells demonstrate differences in chromatin accessibility at key Th1, Th2 and Th17 transcription factor loci. **(a)** Adult naïve CD4^+^ T cells have increased ATAC‐seq peaks at the promoter and sixth exon of the Th1‐associated transcription factor *TBX21* (T‐bet) compared with neonatal cells based on edgeR differential binding. **(b)** Adult naïve CD4^+^ T cells have increased ATAC‐seq peaks at the first intron of the Th2‐associated transcription factor *STAT6* compared with neonatal cells based on edgeR differential binding. **(c)** Neonatal naïve CD4^+^ T cells have increased ATAC‐seq peaks at the promoter of the Th17‐associated transcription factor *STAT3* compared with adult cells based on edgeR differential binding. Adult *n* = 3, neonate *n* = 3, number represents replicate. The black rectangle highlights the differential peaks. ATAC‐seq experiment performed a total of one time. The black arrow indicates gene direction. ATAC‐seq peaks were visualized using the Integrated Genomics Viewer. **(d)** Baseline mRNA expression for the transcription factors *TBX21*, *STAT6* and *STAT3* was compared between RNA‐seq data for naïve CD4^+^ T cells from neonates and adults. RNA‐seq data were obtained from the Blueprint Consortium and differential analysis was performed with DESEQ2. mRNA counts were normalized to RPKM. Neonate *n* = 2, adult *n* = 8. *P < 0.05, ****P < 0.0001. ATAC‐seq, assay for transposase‐accessible chromatin with high‐throughput sequencing; mRNA, messenger RNA; RPKM, reads per kilobase per million; Th, T helper cell. [Colour figure can be viewed at wileyonlinelibrary.com]

Neonatal CD4^+^ T cells demonstrate a Th2 rather than a Th1 bias, with demonstrated deficits during Th17 skewing conditions.^5^, ^16^ Because of this, we evaluated chromatin accessibility at known Th1, Th2 and Th17 cytokines and transcription factors in adult and term healthy neonatal naïve CD4^+^ T cells. Adult cells had increased chromatin accessibility at the Th1‐associated transcription factor *TBX21* (T‐bet) and the Th2‐associated transcription factor *STAT6*, while neonatal cells demonstrated increased chromatin accessibility at the Th17‐associated transcription factor *STAT3* (Table 2, Figure 7a–c). These chromatin accessibility differences were associated with increased *TBX21* and *STAT6* mRNA expression in adult cells and increased *STAT3* mRNA expression in neonatal cells (Figure 7d). No chromatin accessibility differences were noted for the Th1 cytokines *IFNG* and *TNF*, the Th2 cytokines *IL4*, *IL5* and *IL13* or for the Th17 cytokines *IL17A*, *IL17F* and *IL22* (Supplementary figure 4), suggesting that chromatin accessibility changes for these key cytokines occur actively once the cell encounters an appropriate stimulus. Overall, adult naïve CD4^+^ T cells had increased chromatin accessibility at Th1‐ and Th2‐associated transcription factors, while neonatal cells demonstrated increased chromatin accessibility at a Th17‐associated transcription factor, which may contribute to the altered T‐cell phenotypes observed between adult and neonatal cells under different T‐cell polarizing conditions.

**Table 2:** Comparison of ATAC‐seq peaks at Th1‐, Th2‐ and Th17‐specific gene loci between adult and neonatal naïve CD4^+^ T cells

Gene	Increased chromatin accessibility	Location	ATAC‐seq differential binding *P*‐value
Th1			
*STAT1*	Neonate	Promoter	2.2 × 10^−13^
*STAT4*	Adult	Intron 2	0.002
	Adult	Intron 3	0.02
	Neonate	Promoter	0.002
*TBX21* (T‐bet)	Adult	Promoter	0.003
	Adult	Exon 6	0.002
*IFNG*	Not different		
*IFNGR1*	Neonate	Promoter	0.014
*IL12RB1*	Adult	Intron 1	0.002
	Adult	Intron 2	0.0004
*IL12RB2*	Neonate	Intron 7	0.02
	Neonate	Intron 9	7.3 × 10^−7^
*TNF*	Not different		
Th2			
*STAT6*	Adult	Intron 1	0.0003
*GATA3*	Adult	Promoter	0.01
	Neonate	Promoter	0.005
*IL4R*	Adult	Promoter	0.01
	Adult	Intron 1	0.0008
*IL4*	Not different		
*IL5*	Not different		
*IL13*	Not different		
Th17			
*STAT3*	Neonate	Promoter	0.01
*IL23R*	Neonate	Intron 1	0.0002
	Neonate	Intron 3	0.0003
	Neonate	Intron 4	5.3 × 10^−10^
*IL17A*	Not different		
*IL17F*	Not different		
*IL22*	Not different		

ATAC‐seq, assay for transposase‐accessible chromatin with high‐throughput sequencing; Th, T helper cell.

## DISCUSSION

Although neonatal T‐cell responses are crucial for the development of long‐lasting immunity after vaccination and early life pathogen exposures, the mechanisms underlying these responses are poorly understood. In this study, we confirmed a TCR‐dependent activation defect in neonatal naïve CD4^+^ T cells, but demonstrated that neonatal cells were able to robustly activate when stimulated in a TCR‐independent manner. This was associated with decreased mRNA expression from genes associated with TCR signaling but increased mRNA expression from genes associated with alternative modes of activation in the neonatal cells. While there were no global differences in H3K4me3 between neonatal and adult cells, the adult cells did demonstrate increased global chromatin accessibility. In addition, locations with H3K4me3 were more likely to be associated with open chromatin and active gene transcription in the adult cells. Several TCR signaling‐associated gene loci demonstrated increased mRNA expression and enrichment in either H3K4me3 or enhanced chromatin accessibility in adult naïve CD4^+^ T cells, including *FYN*, *ITK*, *CD4*, *LAT* and *LCK*. By contrast, neonatal naïve CD4^+^ T cells demonstrated increased mRNA expression and H3K4me3 enrichment or increased chromatin accessibility at numerous TCR‐independent gene loci, including *NRAS*, *JUN* (AP‐1), *RAF1*, *NFKB1* and *NFKB2*. We additionally found that adult cells had increased chromatin accessibility and mRNA expression at the sites of the Th1‐associated transcription factor *TBX21* (T‐bet) and the Th2‐associated transcription factor *STAT6*, while neonatal cells had increased chromatin accessibility and mRNA expression at the Th17‐associated transcription factor *STAT3*. These findings suggest that chromatin accessibility may underlie differences in naïve CD4^+^ T‐cell activation and skewing between neonates and adults.

Epigenetics, which involves changes to DNA structure and gene expression without altering the underlying genetic code, is increasingly recognized to contribute to immune cell development, differentiation and function.^10^ In neonatal CD4^+^ T cells, hypermethylation of the IL‐2 promoter (suppresses gene transcription) and hypomethylation of the IL‐4 promoter (allows for active gene transcription) is suggested to partially explain the neonatal Th2 bias.^8^ In addition, differences in histone 3 and histone 4 acetylation in umbilical cord blood CD4^+^ T cells have been associated with the development of allergic diseases during childhood.^17^ We have previously demonstrated that monocytes gain the activating histone tail modification H3K4me3 at immunologically relevant promoter locations over the course of development from extremely preterm neonate to adult, and that this gain is associated with more robust monocyte responses.^10^ We are the first group to investigate H3K4me3 patterning and chromatin accessibility in neonatal CD4^+^ T cells, demonstrating that neonatal naïve CD4^+^ T cells have more transcriptionally inactive sites (closed chromatin) and that sites with H3K4me3 enrichment are more likely to be associated with closed chromatin and no mRNA transcription compared with adult cells. Similar to our findings in monocytes, there was H3K4me3 enrichment and/or enhanced chromatin accessibility at genes involved in TCR signaling in adult naïve CD4^+^ T cells that had more robust functional T‐cell responses following TCR engagement. Both neonatal monocytes and neonatal naïve CD4^+^ T cells primarily had H3K4me3 enrichment in developmental pathways, including nervous system and cardiac development, that were either not present or less pronounced in adult monocytes or adult naïve CD4^+^ T cells.^10^ This may suggest that early monocyte and T‐cell baseline H3K4me3 patterning stems from common bone marrow progenitor cells, and that this baseline patterning is set before more terminally differentiated cells are present. More lineage‐specific H3K4me3 patterning would then be directed by cell‐type–specific stimulation and interactions. This is consistent with findings for other immune cells, including macrophages.^18^

Although we have demonstrated differences in naïve CD4^+^ T‐cell H3K4me3 enrichment between neonates and adults, it is unclear what is driving these differences. There are eight known H3K4 methyltransferases that perform trimethylation, although not all of these have been shown to be active in human tissues.^14^ We found that adult naïve CD4^+^ T cells have increased expression of *KMT2A* (*MLL1*) and *SET1A* compared with neonatal cells. Significant redundancy exists between H3K4 methyltransferases, as they are crucial for a wide array of developmental processes.^19^ In addition, some of these H3K4 methyltransferases have known roles in CD4^+^ T‐cell differentiation and function, including KMT2A (*MLL1*) and SMYD3.^20^, ^21^ It is possible that the two H3K4 methyltransferases with enhanced expression in the adult cells are contributing to the developmental differences in H3K4me3 enrichment. However, additional studies are needed to better understand how each individual H3K4 methyltransferase contributes to these differences in CD4^+^ T‐cell H3K4me3 patterning.

Neonatal CD4^+^ T cells exhibit defects in TCR signaling after engagement of the TCR–CD3 complex, which remains unchanged even when the T‐cell co‐receptor CD28 is involved. These defects include decreased cell surface CD69 expression, dampened IL‐2 and IFNγ expression and in some cases cellular apoptosis.^5^, ^6^ Our findings are consistent with these, as naïve neonatal CD4^+^ T cells in this study demonstrated decreased markers of activation (CD69) and decreased IL‐2 and TNF‐α expression after TCR signaling was initiated by anti‐CD3/anti‐CD28 stimulation. This does not appear to be a global activation defect, as neonatal naïve CD4^+^ T cells demonstrated increased IL‐2 and TNF‐α expression following TCR‐independent stimulation with PMA/ionomycin. As a potential explanation for these findings, we demonstrated that neonatal naïve CD4^+^ T cells had either decreased H3K4me3 or diminished chromatin accessibility at multiple genes involved in TCR signaling, including *FYN*, *ITK*, *CD4*, *LAT* and *LCK*. These differences were associated with decreased mRNA expression in the neonatal cells. Deficiencies in *CD4*, *LCK*, *LAT* and *ITK* have all been associated with immunodeficiency syndromes and an increased risk of childhood infections, highlighting their importance in determining infection risk.^22^, ^23^, ^24^ Naïve neonatal CD4^+^ T cells exhibited decreased IFNγ and IL‐17A expression when stimulated either through a TCR‐dependent or through a independent pathway, which is also consistent with what has been reported.^25^, ^26^ This suggests that cell intrinsic differences in neonatal naïve CD4^+^ T cells regulate the production of both IFNγ and IL‐17A. We found decreased chromatin accessibility at the Th1‐associated transcription factor *TBX21* (T‐bet) in neonatal cells as one possible explanation for the cell intrinsic defect in IFNγ expression. Interestingly, we observed increased chromatin accessibility at the Th17‐associated transcription factor *STAT3* in neonatal cells, although they are deficient in IL‐17A expression. This suggests that *STAT3*‐associated pathways may be differentially regulated in neonatal and adult CD4^+^ T cells, but further studies are warranted. Despite the well‐published Th2 bias of neonatal CD4^+^ T cells, we found minimal expression of the Th2‐associated cytokines IL‐4, IL‐5 and IL‐13 in neonatal naïve CD4^+^ T cells 24 h after either anti‐CD3/anti‐CD28 or PMA/ionomycin stimulation. This may be related to differences between murine and human responses as many of the studies demonstrating a neonatal Th2 bias utilize mice.^27^, ^28^ This could also relate to the 24‐h timepoint used in our study to evaluate cytokine protein expression, the fact that we used anti‐CD3/anti‐CD28 beads for stimulation or the method we used to quantitate cytokine expression, as other studies demonstrating a neonatal CD4^+^ T‐cell Th2 bias in human cells used different stimulation times,^29^ anti‐CD3/anti‐CD28 antibodies instead of beads for stimulation^5^ or used either ELISA technology or flow cytometry for protein quantification.^5^, ^29^ Taken together, these findings suggest that differences in H3K4me3 and chromatin accessibility may contribute to the ability of neonatal naïve CD4^+^ T cells to respond to stimulation in both TCR‐dependent and TCR‐independent fashions.

Infections make up a large proportion of potentially preventable deaths during the neonatal and infant periods.^1^ There are currently few available vaccines that are able to stimulate the neonatal immune system to mount effective and long‐lasting responses to vaccine‐preventable pathogens. The primary role of vaccines is to induce B cells to produce functional antibodies against a pathogen. However, induction of IFN‐γ‐producing memory T cells that are able to kill infected cells is becoming an increasingly studied and promising vaccine approach.^30^ The TCR–CD3 complex recognizes antigen bound to major histocompatibility complex molecules on the surface of antigen‐presenting cells and directs T‐cell activation, cytokine expression and memory T‐cell differentiation. An optimal T‐cell vaccine would induce long‐lived pathogen‐specific memory T cells that would be capable of directing pathogen clearance before a severe infection could develop. Given the importance of TCR signaling in IFN‐γ‐producing memory T‐cell differentiation and the significant defects naïve neonatal CD4^+^ T cells demonstrate in TCR signaling, neonatal T‐cell vaccine development has been minimally successful.^31^ The findings of this study provide some mechanistic insight into why naïve neonatal CD4^+^ T cells demonstrate defects in TCR signaling and subsequent activation, which may help guide future immunization strategies.

We recognize that this study has several limitations. Pooling of neonatal samples for ChIP‐seq helped to minimize interpatient variability and genetic diversity, but eliminated the ability to identify patient‐specific differences in T‐cell H3K4me3 patterning. The inclusion of three or four ChIP‐seq and ATAC‐seq replicates per age group is low, but has been shown to be sufficient for site discovery in next‐generation sequencing experiments.^32^ There were small differences in cell purity following negative selection for naïve CD4^+^ T cells between the adult and neonatal groups. While these differences were statistically significant and should be taken into account when considering our findings, we think these differences were unlikely to have major biological relevance or alter the main findings of this study. We only evaluated differences in naïve CD4^+^ T cell H3K4me3 patterning and chromatin accessibility and how these changes were associated with differences in cell function, while it is likely that other factors, including additional histone tail modifications (e.g. H3K4me1, H3K9me3, H3K27me3) and DNA methylation, are also involved. It is also likely that similar chromatin accessibility differences exist in neonatal memory/effector CD4^+^ T cells that would have important implications for vaccine responses and the development of immunological memory, but this was beyond the scope of this study.

## CONCLUSIONS

This study demonstrated that neonatal naïve CD4^+^ T cells had a TCR‐dependent defect in activation but were able to robustly activate when stimulated through a TCR‐independent pathway. This defect in TCR‐associated activation was associated with a decrease in the activating histone tail modification H3K4me3 and/or decreased chromatin accessibility in TCR signaling–associated gene loci in the neonatal cells. These findings increase our understanding of why neonatal naïve CD4^+^ T cells demonstrate defective TCR signaling and subsequent activation, and may have implications for alternative neonatal immunization strategies.

## METHODS

### Study participants

All of the studies were approved by the Iowa Institutional Review Board. This study conforms to the US Federal Policy for the Protection of Human Subjects. Written informed consent was obtained from all adult study participants and from the parents of all neonatal study participants. Neonatal samples were either collected from the University of Iowa Maternal Fetal Tissue Bank^33^ or from the University of Iowa Neonatal Intensive Care Unit Laboratory from February 2021 to April 2022.

### Blood processing

Peripheral blood was collected by venipuncture from healthy adults. Umbilical cord blood from neonatal study participants was collected from the umbilical vein prior to delivery of the placenta during vaginal deliveries and after removal of the placenta in c‐section deliveries. The adult peripheral blood and umbilical cord blood were processed within 24 h of initial collection. Whole blood samples were diluted 1:2 with sterile 0.9% saline (Thermo Fisher Scientific, Waltham, MA, USA) and Ficoll–Isopaque (Thermo Fisher Scientific, Waltham, MA, USA) density gradient centrifugation was used to isolate mononuclear cells. Whole mononuclear cells were then placed in autologous serum with 10% dimethyl sulfoxide (Thermo Fisher Scientific, Waltham, MA, USA ) in cryovials (Corning, Tewksbury, MA, USA) and were frozen at a controlled temperature of −1°C per minute using a Mr. Frosty Freezing Container (Thermo Scientific, Waltham, MA, USA) and either transferred to liquid nitrogen for long‐term storage (samples from the University of Iowa Maternal Fetal Tissue Bank) or kept at −80°C until use (samples from the University of Iowa Neonatal Intensive Care Unit Laboratory). Whole mononuclear cells were washed twice with sterile Roswell Park Memorial Institute (RPMI) (Gibco, Waltham, MA, USA) medium with 10% human serum (Sigma Aldrich, Burlington, MA, USA) after thawing. Naïve CD4^+^ T cells were isolated using negative magnetic bead selection according to the manufacturer's instructions (catalog number 17555; STEMCELL Technologies, Vancouver, BC, Canada). The average viability postfreezing was 99.8% for adults and 95.6% for neonates by flow cytometry as shown in Supplementary figure 5a–d (LIVE/DEAD Fixable Violet; Life Technologies, Carlsbad, CA, USA). The average purity of the CD4^+^ T cells was 99.4% for adults and 98.1% for neonates measured by flow cytometry (Supplementary figure 5e–g) using anti‐human CD4 (clone SK3, PerCP/Cy5.5; BioLegend, San Diego, CA, USA). CD4^+^ T‐cell subsets were evaluated by flow cytometry using the following antibodies: LIVE/DEAD Fixable Violet (Life Technologies), CD3 (clone HIT3a, Alexa Fluor 488; BioLegend), CD4 (clone SK3, PerCP/Cy5.5; BioLegend), CD45RA (clone HI100, APC/Cy7; BioLegend) and CCR7 (clone G043H7, PE/Cy7; BioLegend) at a 1:100 dilution. Approximately 97% of adult and 95% of neonatal CD4^+^ T cells were naïve as shown in Supplementary figure 5h.

### H3K4me3 ChIP‐seq

Naïve CD4^+^ T cells were fixed in 2% paraformaldehyde (Thermo Fisher Scientific, Waltham, MA, USA) immediately after cell purification. Chromatin immunoprecipitation followed by massively parallel DNA sequencing was performed as we have previously published on naïve CD4^+^ T cells from healthy adults and healthy term neonates.^10^, ^13^ The adult age group consisted of three biological replicates and the neonatal age group contained four biologic replicates. Each neonatal replicate contained up to 14 pooled samples to achieve a starting cell concentration of at least 1 × 10^6^ cells per immunoprecipitation (Supplementary table 8). Ultrasonication was performed using the Bioruptor Pico (Diagenode, Denville, NJ, USA) with a 5‐min sonication cycle with 30 s “on” and 30 s “off.” These sonication settings were optimized to obtain 150–400‐bp fragments of DNA. The immunoprecipitation used 4 μg of the ENCODE Consortium–recommended anti‐H3K4me3 antibody (ab8580; Abcam, Cambridge, MA, USA). The samples were pooled in equimolar concentrations following library preparation and 100 nucleotide single‐end sequencing was performed on a NovaSeq 6000 machine (Illumina, San Diego, CA, USA). This resulted in 30–50 million reads/sample. Sample quality was evaluated using FastQC (Version 0.11.9, Simon Andrews, Babraham Bioinformatics, Cambridge, UK)^34^ and ChIPQC (Version 1.32.0, Tom Carroll and Rory Stark, University of Cambridge, Cambridge, UK).^35^ Individual sample quality control measures are detailed in Supplementary table 9. Adapter sequences were trimmed from raw reads using Trimmomatic V0.39 (Usadel Lab, RWTH Aachen University, Aachen, Germany).^36^ Trimmed reads were aligned to the *Homo sapiens* genome assembly (hg19, GRCh37) with Bowtie2 V2.3.4.2 (Ben Langmead, Johns Hopkins University, Baltimore, MD, USA) using default parameters.^37^ Mapped files were converted from SAM to BAM and were sorted using SAMtools V1.3.1 (Heng Li, John Marshall and Petr Danecek, Sanger Institute, Cambridgeshire, UK).^38^ H3K4me3 peaks were called using MACS2 V2.1.1.20160309 (Meeta Mistry and Radhika Khetani, Boston, MA, USA) by comparing immunoprecipitated samples with input samples using default settings.^39^ Peaks that were present in at least two biologic replicates were included in a consensus peakset for each group and were used for downstream analysis. DiffBind (Version 3.6.1, Rory Stark and Gord Brown, University of Cambridge, Cambridge, UK) was used to compare H3K4me3 peaks between different groups after reads were normalized to total read count and library size.^40^ Differentially bound H3K4me3 peaks were identified using edgeR V3.38.0 (Yunshun Chen and Gordon Smyth, Walter and Eliza Hall Institute of Medical Research, Parkville, Australia).^40^ The false discovery rate threshold for differentially bound sites was set to < 0.05. ChIPseeker (Version 1.32.0, Guangchuang Yu, Southern Medical University, Guangzhou, Guangdong, China) was used to annotate peaks.^41^ ClusterProfiler (Version 4.4.2, Guangchuang Yu, Southern Medical University, Guangzhou, Guangdong, China) was used to determine gene ontology over‐representation, and the *P*‐values were adjusted for multiple comparisons.^42^ Data visualization was performed using the Integrated Genomics Viewer V2.4.16 (Broad Institute, Cambridge, MA, USA).^43^ The ChIP‐seq data sets included in this manuscript are available in the Gene Expression Omnibus (GEO) repository GSE202904.

### ATAC‐seq

ATAC‐seq was performed following cell purification as previously published.^44^ Each age group consisted of three biological replicates and contained 50 000 cells (Supplementary table 10). In brief, 50 000 naïve CD4^+^ T cells underwent transposition at 37°C for 30 min using 2.5 μL of the Nextera Tn5 Transposase (Illumina, San Diego, CA, USA). The samples were then purified using a Qiagen MinElute PCR Purification Kit (Qiagen, Germantown, MD, USA) and were eluted in 10 μL of elution buffer (10 mm Tris buffer, pH 8). The transposed DNA was amplified using custom Nextera PCR primers.^44^ The amplified libraries were purified using a Qiagen MinElute PCR Purification Kit (Qiagen) and were eluted in 20 μL of elution buffer (10 mm Tris buffer, pH 8). The samples were pooled in equimolar concentrations following library preparation and 200‐nucleotide paired‐end sequencing was performed on a NovaSeq 6000 (Illumina, San Diego, CA, USA) machine. This resulted in 50–70 million reads/sample. Sample quality was evaluated using FastQC (Version 0.11.9, Simon Andrews, Babraham Bioinformatics, Cambridge, UK)^34^ and ChIPQC (Version 1.32.0, Tom Carroll and Rory Stark, University of Cambridge, Cambridge, UK).^35^ Individual sample quality control measures are detailed in Supplementary table 11. Paired‐end .fastq files were trimmed using Trimmomatic V0.39 (Usadel Lab, RWTH Aachen University, Aachen, Germany).^36^ Trimmed files were aligned to the GRCh37 genome assembly using Bowtie2 V2.3.4.2 (Ben Langmead, Johns Hopkins University, Baltimore, MD, USA) with default settings.^37^ Mapped files were converted from SAM to BAM and were sorted using SAMtools V1.3.1 (Heng Li, John Marshall and Petr Danecek, Sanger Institute, Cambridgeshire, UK).^38^ Peaks were called using MACS2 V2.1.1.20160309 (Meeta Mistry and Radhika Khetani, Boston, MA, USA) using default parameters.^39^ Peaks that were present in at least two biologic replicates were included in a consensus peakset for each group and were used for downstream analysis. DiffBind (Version 3.6.1, Rory Stark and Gord Brown, University of Cambridge, Cambridge, UK) was used to compare ATAC‐seq peaks between different groups after reads were normalized to total read count and library size.^40^ Differentially bound peaks were identified using edgeR V3.38.0 (Yunshun Chen and Gordon Smyth, Walter and Eliza Hall Institute of Medical Research, Parkville, Australia).^40^ The false discovery rate threshold for differentially bound sites was set to < 0.05. ChIPseeker (Version 1.32.0, Guangchuang Yu, Southern Medical University, Guangzhou, Guangdong, China) was used to annotate peaks.^41^ Data visualization was performed using the Integrated Genomics Viewer V2.4.16 (Broad Institute, Cambridge, MA, USA).^43^ The ATAC‐seq data sets included in this manuscript are available in the Gene Expression Omnibus (GEO) repository GSE202905.

### RNA‐seq

This study makes use of data generated by the Blueprint Consortium. A full list of the investigators who contributed to the generation of the data is available from www.blueprint‐epigenome.eu. Funding for the project was provided by the European Union's Seventh Framework Programme (FP7/2007–2013) under grant agreement no 282510 – BLUEPRINT. The Blueprint data set included in this manuscript is EGAD00001002348, which included RNA‐seq data for CD3^+^CD4^+^CD45RA^+^ alpha‐beta T cells (naïve) from two healthy term neonates and eight healthy adults. These samples were analyzed from raw reads. Paired‐end .fastq files were trimmed using Trimmomatic V0.39 (Usadel Lab, RWTH Aachen University, Aachen, Germany).^36^ Trimmed files were aligned to the GRCh37 genome assembly using Bowtie2 V2.3.4.2 (Ben Langmead, Johns Hopkins University, Baltimore, MD, USA) with default settings.^37^ Mapped files were converted from SAM to BAM and sorted using SAMtools V1.3.1 (Heng Li, John Marshall and Petr Danecek, Sanger Institute, Cambridgeshire, UK).^38^ Rsubread V2.10.0 (Wei Shi and Yang Liao, Olivia Newton John Cancer Research Institute, Heidelberg, Australia and Gordon Smyth, Walter and Eliza Hall Institute of Medical Research, Parkville, Australia) was used to align reads, quantify transcript abundance and assign reads to genomic features.^45^ Reads were normalized to total read counts and differentially expressed transcripts were identified using the DESEQ2 package V1.36.0 (Michael Love, Genome Biology Unit, European Molecular Biology Laboratory, Heidelberg, Germany).^46^ Normalized read counts were converted to reads per kilobase per million using the edgeR package V3.38.0 (Yunshun Chen and Gordon Smyth, Walter and Eliza Hall Institute of Medical Research, Parkville, Australia).^40^ The false discovery rate for differentially expressed transcripts was set to < 0.05. Genes were annotated using MyGene V1.32.0 (Adam Mark, Cyrus Afrasiabi and Chunlei Wu, Scripps Research Institute, La Jolla, CA, USA).^47^

### Cell culture and protein measurement

Naïve CD4^+^ T cells were plated at a concentration of 1 × 10^5^ cells/well in a flat‐bottomed 96‐well polystyrene cell culture plate (Corning, Tewksbury, MA, USA) in 200 μL of Roswell Park Memorial Institute (RPMI) (Gibco, Waltham, MA, USA) media containing 400 mm/L l‐glutamine (Thermo Fisher Scientific, Waltham, MA, USA), 10% adult human AB^−^ serum (Sigma Aldrich, Burlington, MA, USA), 1% penicillin/streptomycin (Thermo Fisher Scientific, Waltham, MA, USA), 1% sodium pyruvate (Thermo Fisher Scientific, Waltham, MA, USA) and 1% non‐essential amino acids (Thermo Fisher Scientific, Waltham, MA, USA). The cells were either left unstimulated, were stimulated with Dynabeads Human T‐Activation anti‐CD3/anti‐CD28 beads 2 μL per well (Gibco, Waltham, MA, USA) or were stimulated with PMA 25 ng mL^−1^ (P1585; Millipore Sigma, Burlington, MA, USA) and ionomycin 1 μg mL^−1^ (IO634; Millipore Sigma) and were incubated at 37°C with 5% CO_2_. Cells were collected for flow cytometry 5 h after plating for unstimulated cells, 5 h after stimulation with PMA and ionomycin and 48 h after stimulation with anti‐CD3 and anti‐CD28. Cell culture supernatants were collected 24 h after plating/stimulation for all conditions. Protein levels of the cytokines IL‐2, IL‐4, IL‐5, IL‐13, IL‐17A, TNF‐α and IFN‐γ were measured from cell culture supernatants by a Bio‐Plex bead–based multiplex assay on a Bio‐Plex 200 Machine following the manufacturer's instructions (Bio‐Rad, Hercules, CA, USA). Phosphorylated levels of CD3ε and ERK were measured by a MILLIPLEX MAP T‐Cell Receptor Magnetic Bead Kit (Millipore Sigma) on a Bio‐Plex 200 Machine following the manufacturer's instructions (Bio‐Rad).

### Flow cytometry

Upon collection from the cell culture plate, naïve CD4^+^ T cells were resuspended in (Thermo Fisher Scientific, Waltham, MA, USA) phosphate‐buffered saline with 0.002 m (Thermo Fisher Scientific, Waltham, MA, USA) ethylenediaminetetraacetic acid and 1% (Millipore Sigma, Burlington, MA, USA) fetal calf serum (flow buffer). Cells were stained for viability using LIVE/DEAD Fixable Violet at a 1:50 dilution (Life Technologies). Cells were washed twice with flow buffer and CD69 (clone FN50, APC/Cy7; BioLegend) and CD4 (clone SK3, PerCP/Cy5.5; BioLegend) were labeled using antibodies at a 1:100 dilution. Cells were labeled according to the manufacturer's directions. Flow cytometry was performed on a CytoFLEX machine with 405‐, 488‐ and 638‐nm lasers (Beckman Coulter, Brea, CA, USA). The gating strategy is shown in Supplementary figure 6. FlowJo version 10 (Becton Dickinson & Company, Franklin Lakes, NJ, USA) was used for compensation, gating and data visualization.

### mRNA

Cellular mRNA was extracted and purified from unstimulated naïve CD4^+^ T cells immediately following negative selection using an RNeasy Micro Kit (Qiagen) according to the manufacturer's instructions. Complementary DNA was synthesized with 50 ng of RNA using the iScript cDNA Synthesis Kit (Bio‐Rad) following the manufacturer's instructions. Real‐time quantitative PCR was performed using TaqMan Primers with an fluorescein‐conjugated probe and Universal PCR reagent (Applied Biosystems, Bedford, MA, USA) to measure levels of *FYN*, *KMT2A*, *KMT2B*, *KMT2C*, *KMT2D*, *SET1A*, *SET1B* and *SMYD3*. The housekeeping gene *ACTB* β‐actin (primer sequences listed in Supplementary table 12) was measured using SYBR Green (Thermo Fisher Scientific, Waltham, MA, USA). mRNA levels were normalized to the housekeeping gene *ACTB* β‐actin and compared using the ΔCT method. Reactions were run on a Bio‐Rad CFX Connect Real‐Time System (Bio‐Rad).

### Statistical analysis

Prism 8 (Graphpad Prism, LLC, San Diego, CA, USA) was used for basic data analysis. Normality of data was assessed using the Shapiro–Wilk test. Differences between groups were evaluated with the Student's *t*‐test for parametric quantitative data, the Mann–Whitney *U*‐test for nonparametric quantitative data, ANOVA with Holm–Šídák multiple comparisons correction for multiple comparisons of parametric data, the Kruskal–Wallis test with Dunn's multiple comparison correction for multiple comparisons of nonparametric data and Fisher's exact test to evaluate relationships between categorical variables. *P* < 0.05 was considered to be significant.

## AUTHOR CONTRIBUTIONS

**Jennifer Rachelle Bermick:** Conceptualization; data curation; formal analysis; funding acquisition; investigation; methodology; validation; visualization; writing – original draft; writing – review and editing. **Priya Issuree:** Conceptualization; formal analysis; methodology; visualization; writing – review and editing. **Aaron denDekker:** Formal analysis; investigation; methodology; validation; visualization; writing – review and editing. **Katherine Gallagher:** Formal analysis; investigation; methodology; supervision; writing – review and editing. **Donna Santillan:** Methodology; resources; writing – review and editing. **Steven Kunkel:** Formal analysis; investigation; resources; supervision; writing – review and editing. **Nicholas Lukacs:** Conceptualization; formal analysis; methodology; resources; supervision; writing – review and editing. **Matthew Schaller:** Conceptualization; formal analysis; investigation; methodology; supervision; visualization; writing – review and editing.

## CONFLICT OF INTEREST

None of the authors have conflicts of interest to report.

## Data Availability

The ChIP‐seq datasets included in this manuscript are available in the GEO repository GSE202904. The ATAC‐seq datasets included in this manuscript are available in the GEO repository GSE202905. This study makes use of data generated by the Blueprint Consortium, and includes dataset EGAD00001002348 with permission of the Blueprint Consortium. The remaining data that support the findings of this study are available from the corresponding author upon reasonable request.
