# Supplementary material for: Differences in H3K4me3 and chromatin accessibility contribute to altered T‐cell receptor signaling in neonatal naïve CD4 T cells
Source: Immunol Cell Biol. 2022 Jun 20;100(7):562–79. doi: 10.1111/imcb.12561 (PMC9357221; doi:10.1111/imcb.12561)
Supplement: Supplementary file 1 [file IMCB-100-562-s005.pdf]

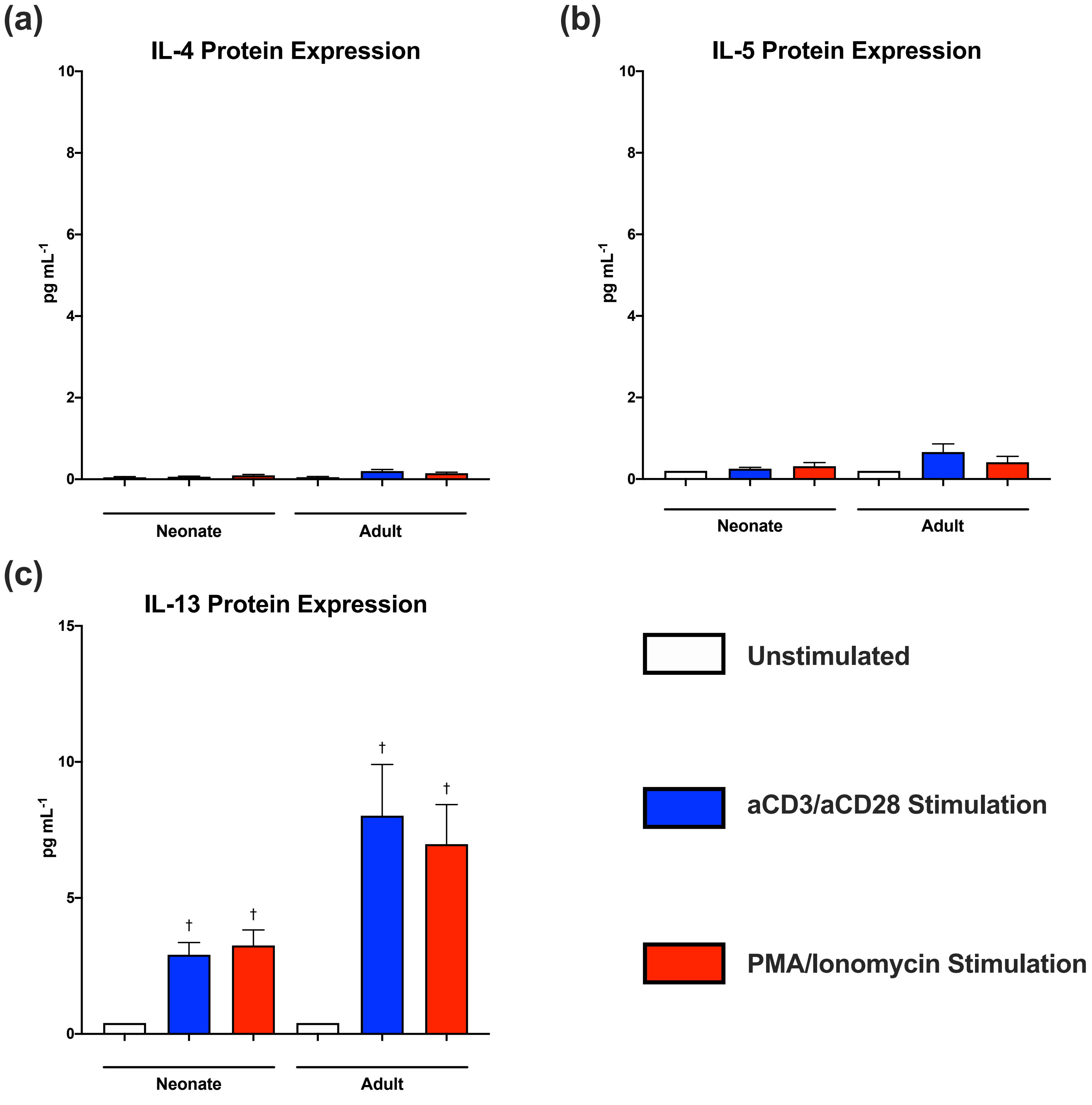

Supplementary figure 1: Adult and neonatal naïve CD4$^+$ T cells demonstrate minimal Th2 cytokine expression following T cell receptor dependent and independent stimulation. A bead-based multiplex assay was used to measure cytokine protein levels in cell culture supernatants from naïve CD4$^+$ T cells from adults and healthy term neonates. Protein expression was measured 24 hours after plating for unstimulated cells, cells stimulated with anti-CD3/anti-CD28 beads or cells stimulated with PMA (25 ng/mL) and ionomycin (1 μg/mL). **(a)** IL-4 protein expression, **(b)** IL-5 protein expression and **(c)** IL-13 protein expression. Experiment was performed a total of three times. Neonate Unstimulated n=5, Neonate aCD3/aCD28 stimulation n=19, Neonate PMA/Ionomycin stimulation n=5, Adult Unstimulated n=10, Adult aCD3/aCD28 stimulation n=11, Adult PMA/Ionomycin stimulation n=9. Box represents mean, error bars represent SEM. Differences between groups were measured with the Kruskal-Wallis test with Dunn's multiple comparisons test. †*P*-value < 0.05 compared to unstimulated control.

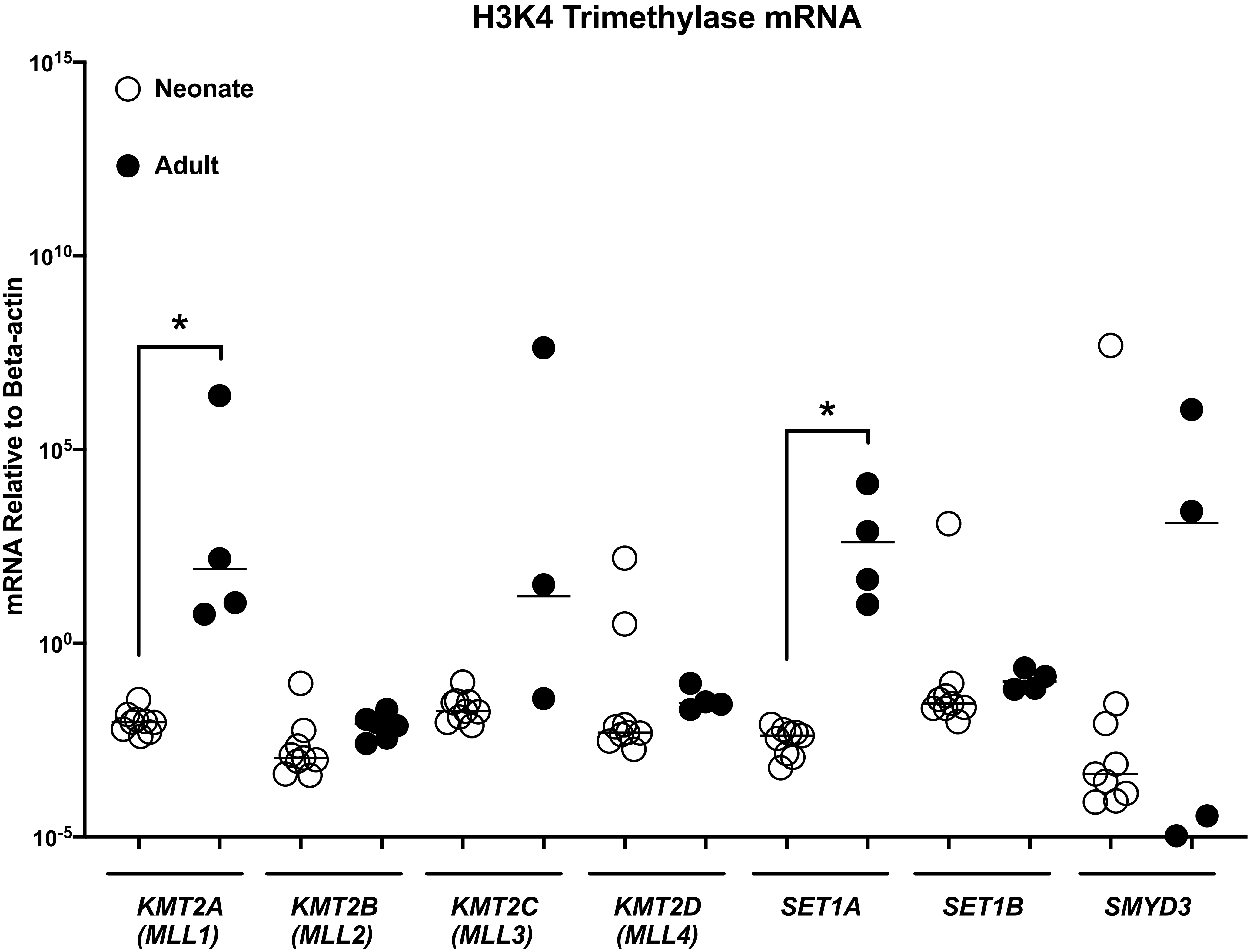

Supplementary figure 2: Adult naïve CD4+ T cells demonstrate increased expression of multiple H3K4me3 methyltransferases compared to neonatal cells. mRNA expression of the H3K4 trimethylases *KMT2A-D*, *SET1A-B* and *SMYD3* were compared between unstimulated adult and healthy term neonatal naïve CD4+ T cells. This experiment was performed a total of two times. Neonate n=9, Adult n=4. Individual data points are shown and the bar represents median. mRNA levels were normalized to the housekeeping gene beta-actin and compared using the ΔCT method. Differences between groups were measured with the Kruskal-Wallis test with Dunn's multiple comparisons test. *P-value < 0.05.

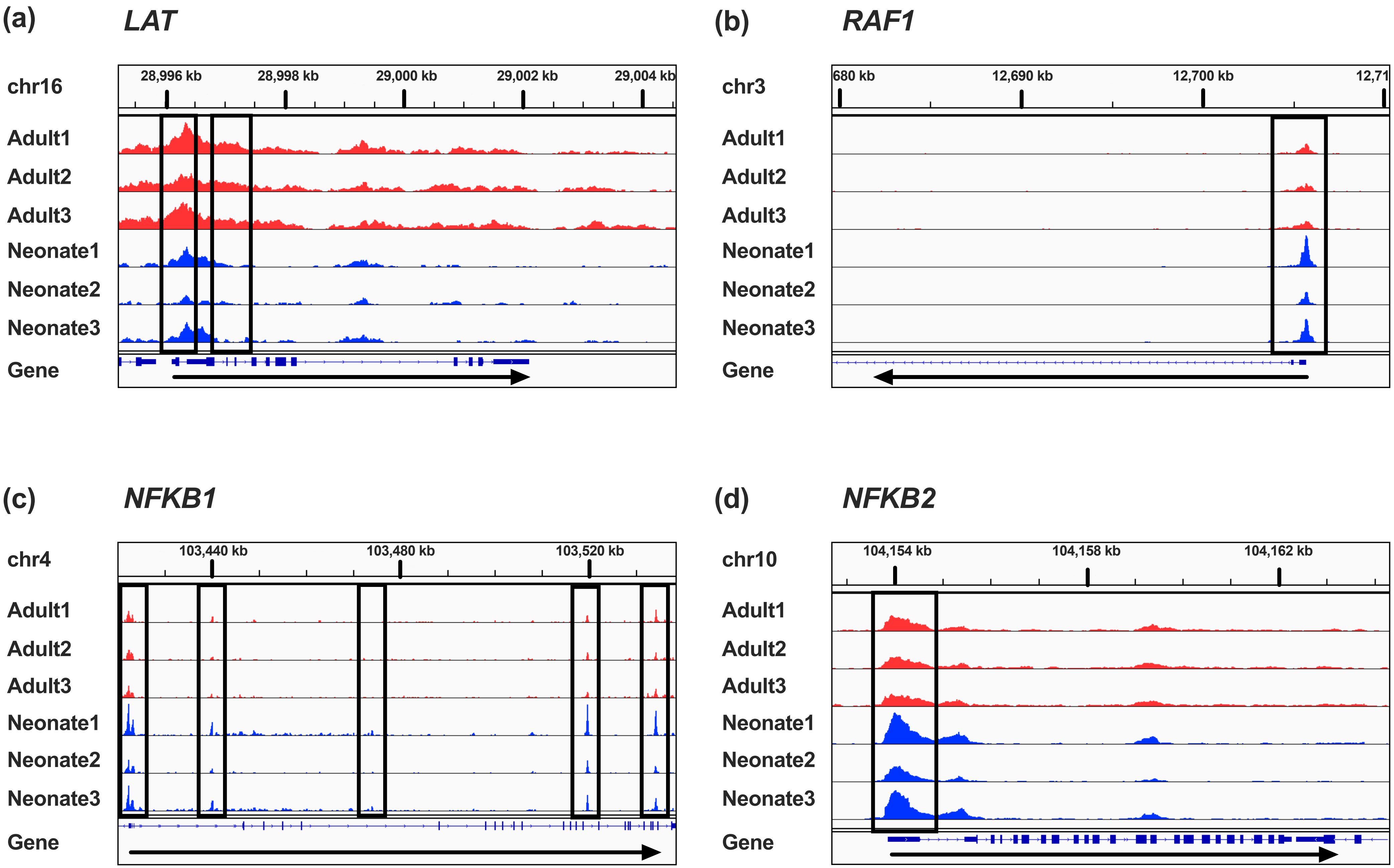

Supplementary figure 3: Differences in chromatin accessibility at T cell activation gene loci between adult and neonatal naive CD4^{+} T cells. **(a)** Adult naïve CD4^{+} T cells have increased ATAC-seq peaks at the promoter and second intron of the T cell signaling gene *LAT* (Linker for activation of T cells) compared to neonatal cells based on edgeR differential binding. **(b)** Neonatal naïve CD4^{+} T cells have increased ATAC-seq peaks at the promoter the common T cell activation gene *RAF1* (Raf1 proto-oncogene, serine/threonine kinase) compared to adult cells based on edgeR differential binding. **(c)** Neonatal naïve CD4^{+} T cells have increased ATAC-seq peaks at the promoter and several introns of the common T cell activation gene *NFKB1* compared to adult cells based on edgeR differential binding. **(d)** Neonatal naïve CD4^{+} T cells have increased ATAC-seq peaks at the promoter the common T cell activation gene *NFKB2* compared to adult cells based on edgeR differential binding. Adult n=3, Neonate n=3, number represents replicate. ATAC-seq experiment performed a total of one time. Black rectangles highlight the differential peaks. Black arrows indicates gene direction. ATAC-seq peaks were visualized using the Integrated Genomics Viewer.

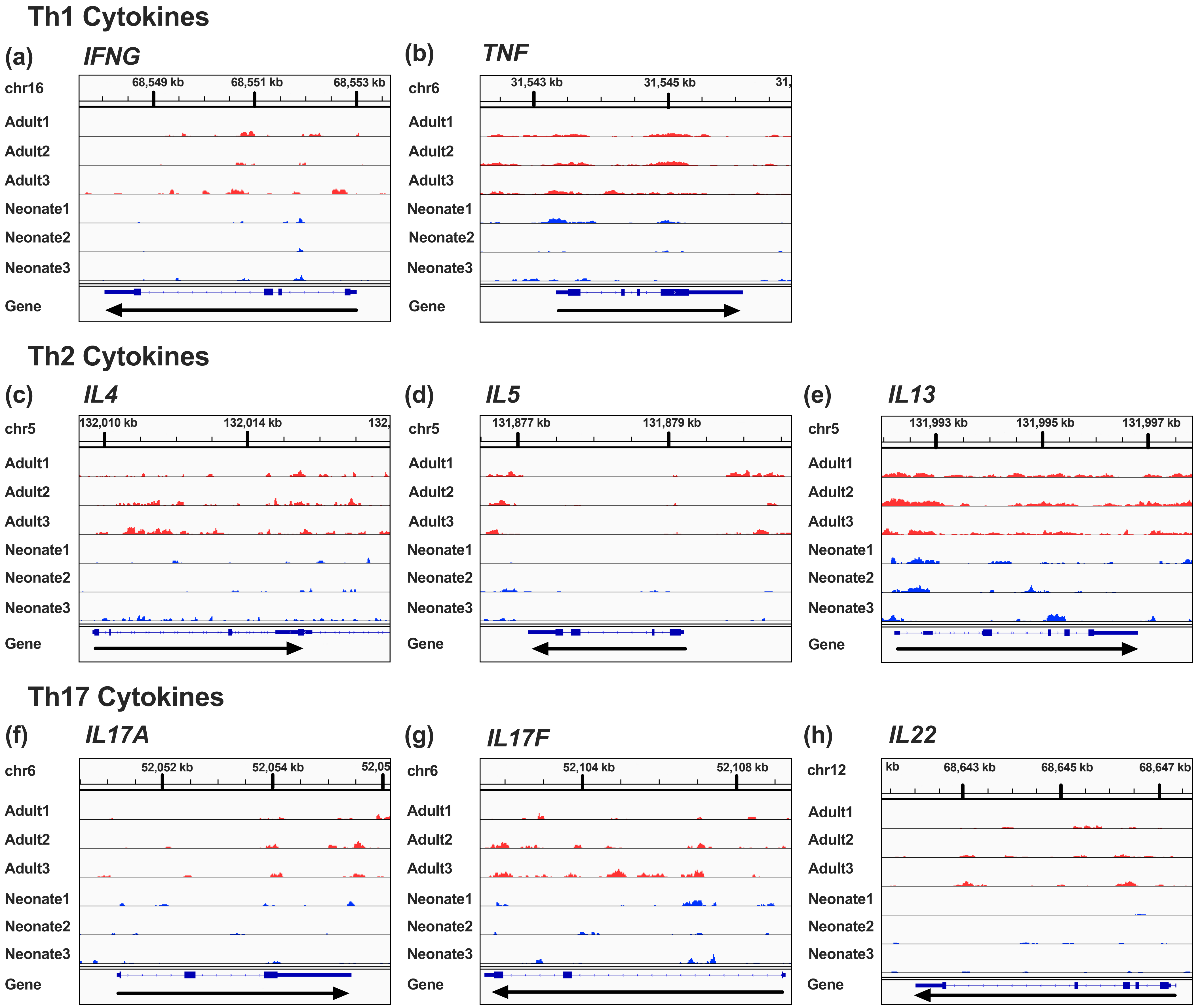

Supplementary figure 4: Neonatal and adult naïve CD4^{+} T cells demonstrate no differences in chromatin accessibility at key Th1, Th2 and Th17 cytokine loci. Visualization of ATAC-seq peaks between adult and neonatal naive CD4^{+} T cells at the Th1 cytokines **(a)** *IFNG* and **(b)** *TNF*, the Th2 cytokines **(c)** *IL4*, **(d)** *IL5* and **(e)** *IL13* and the Th17 cytokines **(f)** *IL17A*, **(g)** *IL17F* and **(h)** *IL22*. Adult n=3, Neonate n=3, number represents replicate. ATAC-seq experiment performed a total of one time. Black arrow indicates gene direction. Differential expression analysis was performed using edgeR. ATAC-seq peaks were visualized using the Integrated Genomics Viewer.

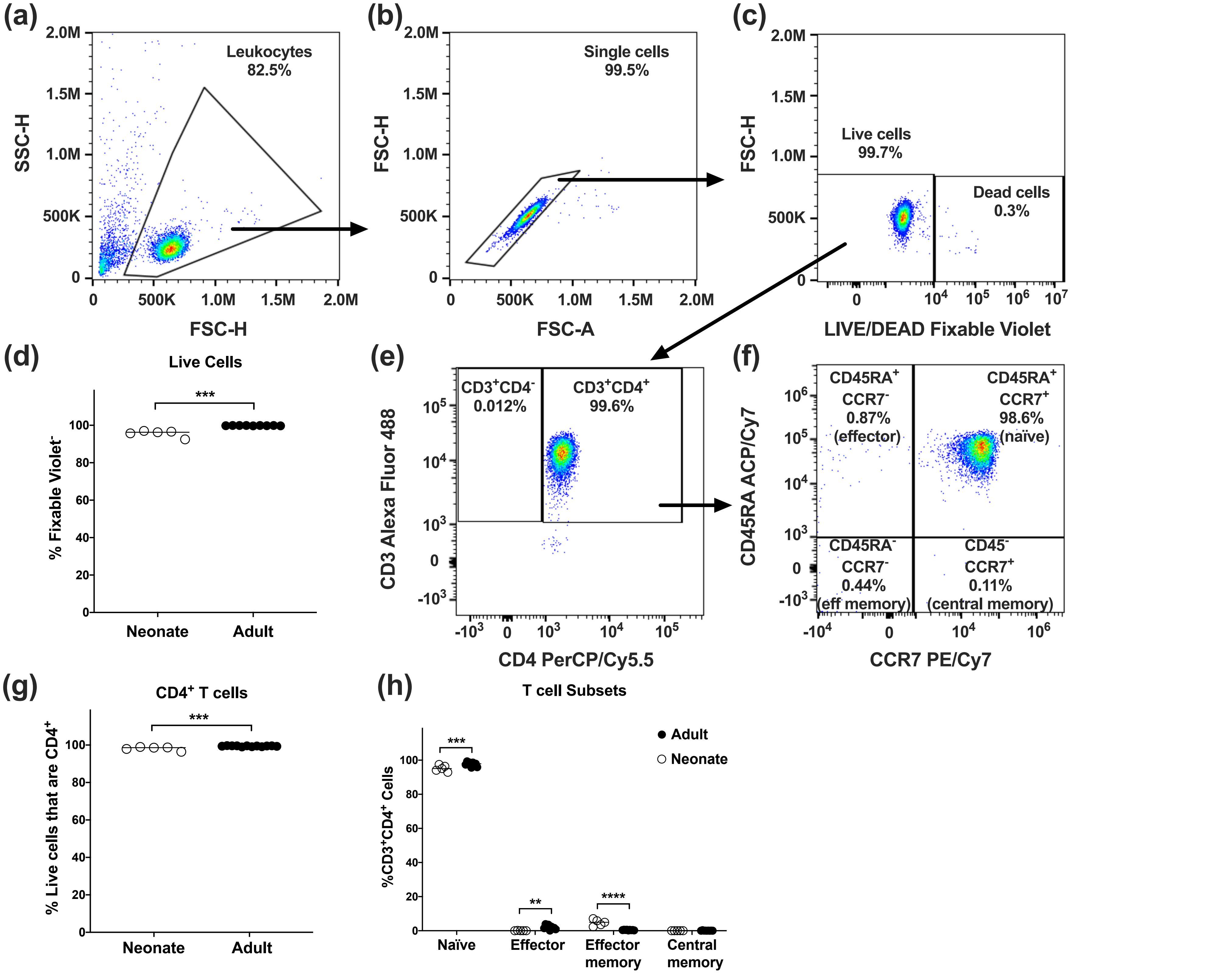

Supplementary figure 5: Flow cytometry gating strategy for markers of T cell subsets**.** T cells were analyzed by flow cytometry. **(a)** Separation of leukocytes from debris. **(b)** Selection of singlets. **(c)** Selection of live cells using Fixable Violet. **(d)** Quantification of live cells between adult and term neonatal cells. This experiment was performed a total of two times. Neonate n=5, Adult n=9. ****P*-value < 0.001. Statistical analysis by Mann-Whitney test. **(e)** Identification of $CD3^+CD4^+$ cells. **(f)** Identification of naïve T cells ($CD45RA^+CCR7^+$), effector T cells ($CD45RA^+CCR7^-$), effector memory T cells ($CD45RA^-CCR7^-$) and central memory T cells ($CD45RA^-CCR7^+$) within the $CD3^+CD4^+$ gate. **(g)** Quantification of $CD4^+$ cells within the live cell gate. This experiment was performed a total of two times. Neonate n=5, Adult n=9. ****P*-value < 0.001. Statistical analysis by unpaired t-test. **(h)** Quantification of naïve, effector, effector memory and central memory T cells within the $CD3^+CD4^+$ gate. This experiment was performed a total of two times. Neonate n=5, Adult n=9. *****P*-value < 0.0001, ****P*-value < 0.001, ***P*-value < 0.01. Statistical analysis by Kruskall-Wallis test with Dunn's Multiple Comparisons.

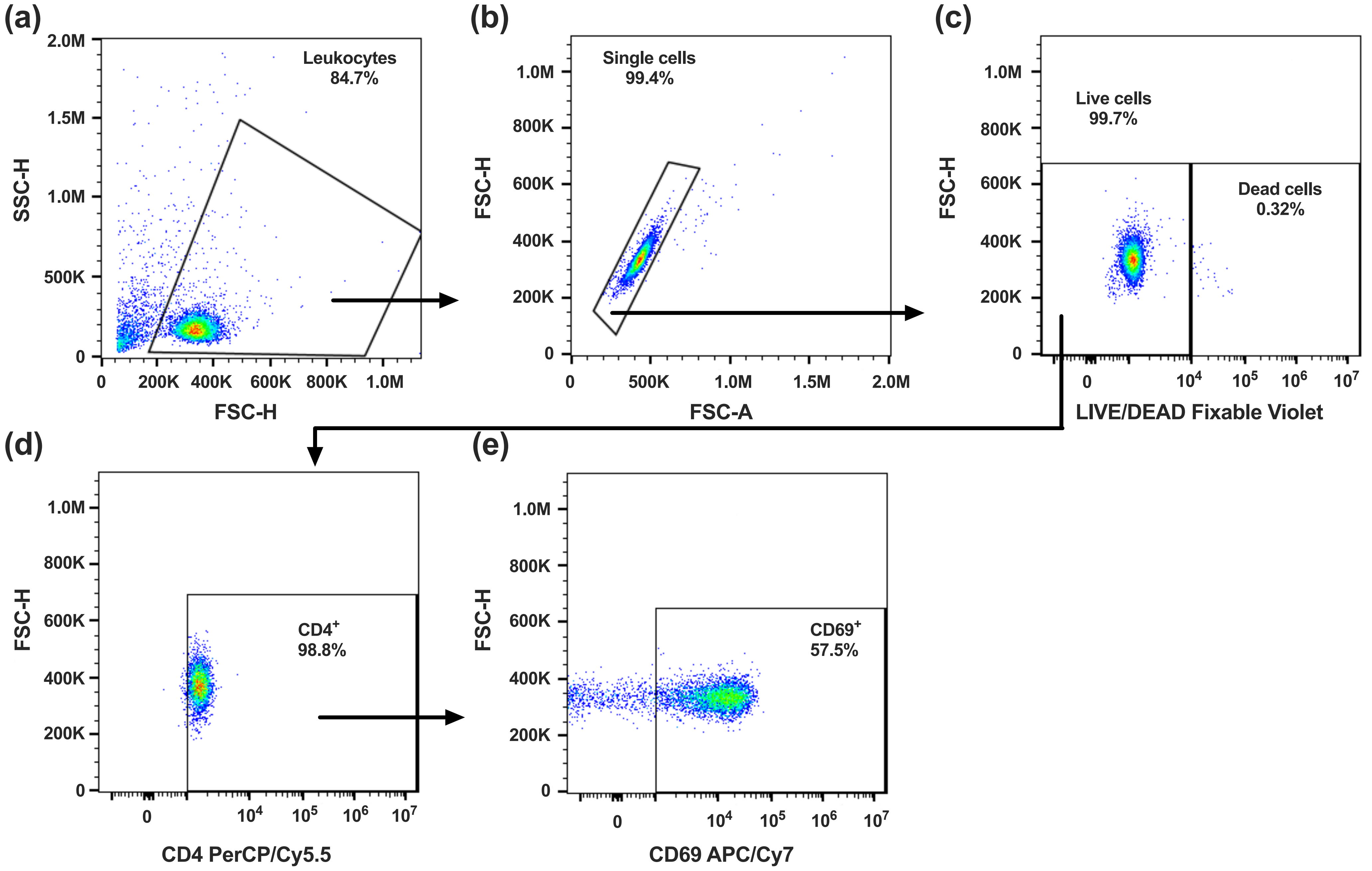

Supplementary figure 6: Flow cytometry gating strategy for markers of T cell activation. T cells were analyzed by flow cytometry. **(a)** Separation of cells from debris. **(b)** Selection of singlets. **(c)** Selection of live cells using Fixable Violet. **(d)** Identification of $CD4^+$ cells. **(e)** Identification of $CD69^+$ cells within the $CD4^+$ gate. This experiment was performed a total of two times.
