# Supplementary material for: Differences in H3K4me3 and chromatin accessibility contribute to altered T‐cell receptor signaling in neonatal naïve CD4 T cells
Source: Immunol Cell Biol. 2022 Jun 20;100(7):562–79. doi: 10.1111/imcb.12561 (PMC9357221; doi:10.1111/imcb.12561)
Supplement: Supplementary file 9 [file IMCB-100-562-s003.docx]

**Supplementary table 8:** ChIP-seq study participants

| **Group** | **Replicate** | **Age** | **Sex** | **# of cells used in ChIP** |
| --- | --- | --- | --- | --- |
| Neonate1 | 1 | Term, 14 pooled samples | Male and Female | 1.7x10^6^ |
| Neonate2 | 2 | Term, 12 pooled samples | Male and Female | 1.3x10^6^ |
| Neonate3 | 3 | Term, 14 pooled samples | Male and Female | 1.4x10^6^ |
| Neonate4 | 4 | Term, 11 pooled samples | Male and Female | 2x10^6^ |
| Adult1 | 1 | 39 years | Female | 1.5x10^6^ |
| Adult2 | 2 | 28 years | Female | 1.5x10^6^ |
| Adult3 | 3 | 39 years | Male | 1.7x10^6^ |

**Supplementary table 9:** H3K4me3 ChIP-seq quality control data

| Sample | Sequencing depth (reads) | Reads Mapped (%) | ^†^SSD | ^‡^FRiP (%) |
| --- | --- | --- | --- | --- |
| Neonate1 | 51,387,591 | 100 | 23.2 | 4 |
| Neonate2 | 43,933,723 | 100 | 22.9 | 4 |
| Neonate3 | 52,486,644 | 100 | 24.5 | 3 |
| Neonate4 | 43,337,707 | 100 | 27.7 | 6 |
| Adult1 | 41,862,365 | 100 | 18.0 | 5 |
| Adult2 | 33,891,010 | 100 | 21.3 | 5 |
| Adult3 | 42,475,982 | 100 | 16.7 | 3 |

^†^SSD is the standard deviation of signal pile-up along the genome normalized to the total number of reads. Values greater than 2 indicate very good enrichment.

^‡^ FRiP is fraction of all mapped reads that fall into peak regions. A FRiP of 1% or greater indicates appropriate enrichment.

**Supplementary table 10:** ATAC-seq study participants

| **Group** | **Replicate** | **Age** | **Sex** | **# of cells used in ATAC-seq** |
| --- | --- | --- | --- | --- |
| Neonate1 | 1 | Term | Female | 50,000 |
| Neonate2 | 2 | Term | Male | 50,000 |
| Neonate3 | 3 | Term | Female | 50,000 |
| Adult1 | 1 | 39 years | Male | 50,000 |
| Adult2 | 2 | 30 years | Male | 50,000 |
| Adult3 | 3 | 39 years | Female | 50,000 |

**Supplementary table 11:** ATAC-seq quality control data

| Sample | Sequencing depth (reads) | Reads Mapped (%) | ^†^SSD | ^‡^FRiP (%) |
| --- | --- | --- | --- | --- |
| Neonate1 | 63,794,606 | 100 | 85.0 | 13 |
| Neonate2 | 52,309,166 | 100 | 51.3 | 9 |
| Neonate3 | 66,966,024 | 100 | 40.6 | 9 |
| Adult1 | 68,456,326 | 100 | 32.2 | 7 |
| Adult2 | 67,547,898 | 100 | 29.6 | 7 |
| Adult3 | 77,555,354 | 100 | 30.4 | 6 |

^†^SSD is the standard deviation of signal pile-up along the genome normalized to the total number of reads. Values greater than 2 indicate very good enrichment.

^‡^ FRiP is fraction of all mapped reads that fall into peak regions. A FRiP of 1% or greater indicates appropriate enrichment.

**Supplementary table 12:** mRNA housekeeping gene primer sequences

| **Primer Name** | **Sequence** |
| --- | --- |
| Beta-actin forward primer | GACGACATGGAGAAAATCTG |
| Beta-actin reverse primer | ATGATCTGGGTCATCTTCTC |
